# Self-Organizing Circuit Assembly through Spatiotemporally Coordinated Neuronal Migration within Geometric Constraints

**DOI:** 10.1371/journal.pone.0028156

**Published:** 2011-11-22

**Authors:** Yi Sun, Zhuo Huang, Kaixuan Yang, Wenwen Liu, Yunyan Xie, Bo Yuan, Wei Zhang, Xingyu Jiang

**Affiliations:** 1 Chinese Academy of Sciences (CAS) Key Lab for Biological Effects of Nanomaterials and Nanosafety, National Center for NanoScience and Technology, Beijing, China; 2 Graduate School of the Chinese Academy of Sciences, Beijing, China; Tel Aviv University, Israel

## Abstract

**Background:**

Neurons are dynamically coupled with each other through neurite-mediated adhesion during development. Understanding the collective behavior of neurons in circuits is important for understanding neural development. While a number of genetic and activity-dependent factors regulating neuronal migration have been discovered on single cell level, systematic study of collective neuronal migration has been lacking. Various biological systems are shown to be self-organized, and it is not known if neural circuit assembly is self-organized. Besides, many of the molecular factors take effect through spatial patterns, and coupled biological systems exhibit emergent property in response to geometric constraints. How geometric constraints of the patterns regulate neuronal migration and circuit assembly of neurons within the patterns remains unexplored.

**Methodology/Principal Findings:**

We established a two-dimensional model for studying collective neuronal migration of a circuit, with hippocampal neurons from embryonic rats on Matrigel-coated self-assembled monolayers (SAMs). When the neural circuit is subject to geometric constraints of a critical scale, we found that the collective behavior of neuronal migration is spatiotemporally coordinated. Neuronal somata that are evenly distributed upon adhesion tend to aggregate at the geometric center of the circuit, forming mono-clusters. Clustering formation is geometry-dependent, within a critical scale from 200 µm to approximately 500 µm. Finally, somata clustering is neuron-type specific, and glutamatergic and GABAergic neurons tend to aggregate homo-philically.

**Conclusions/Significance:**

We demonstrate self-organization of neural circuits in response to geometric constraints through spatiotemporally coordinated neuronal migration, possibly via mechanical coupling. We found that such collective neuronal migration leads to somata clustering, and mono-cluster appears when the geometric constraints fall within a critical scale. The discovery of geometry-dependent collective neuronal migration and the formation of somata clustering *in vitro* shed light on neural development *in vivo*.

## Introduction

The brain is composed of hundreds of nuclei densely populated with neuronal somata, while the rest is packed with interconnecting neurites. The assembly of the neural circuits *in vivo* is achieved through a cascade of processes involving neuronal migration to define the somata locations of neurons[1], [2], [3], [4].

A range of molecular[5] and activity-dependent[6] factors have been elucidated in regulating neuronal migration, mostly at the single cell level[7]. As neurons are dynamically connected with each other through neurite adhesion during development, the migratory behaviors of adjacent neurons within a circuit are coupled, making it a dynamic system. In this regard, systematic analysis of neuronal migration and circuit assembly has been lacking. As a variety of coupled biological systems give rise to emergent self-organization [8], [9], [10], we wondered if self-organization also exists in collective neuronal migration and circuit assembly.

Understanding the collective behavior of neuronal migration and its regulation through geometric constraints may be an important step in understanding circuit assembly in the complex settings of the brain. Geometric constraints can regulate collective behavior of some coupled biological systems[11], we also wondered if the same case takes place in neural circuits.

Neurons and networks in culture have been widely employed for studying formation and function of the nervous systems recently[12], [13], [14], [15], [16]. We set out to study collective neuronal migration and circuit assembly using an *in vitro* model system with dissociated neuronal culture.

## Results

### Neuronal migration at specific developmental stage on coated surfaces *in vitro*

Primary hippocampal neurons from Sprague Dawely (SD) rats (See **Materials and Methods** for details on primary neuron culture) migrate actively on Matrigel (MG) coated gold substrates (gold substrate was used for assembling SAMs throughout the paper, see **Materials and Methods** for details on SAMs).

**Figure 1:**
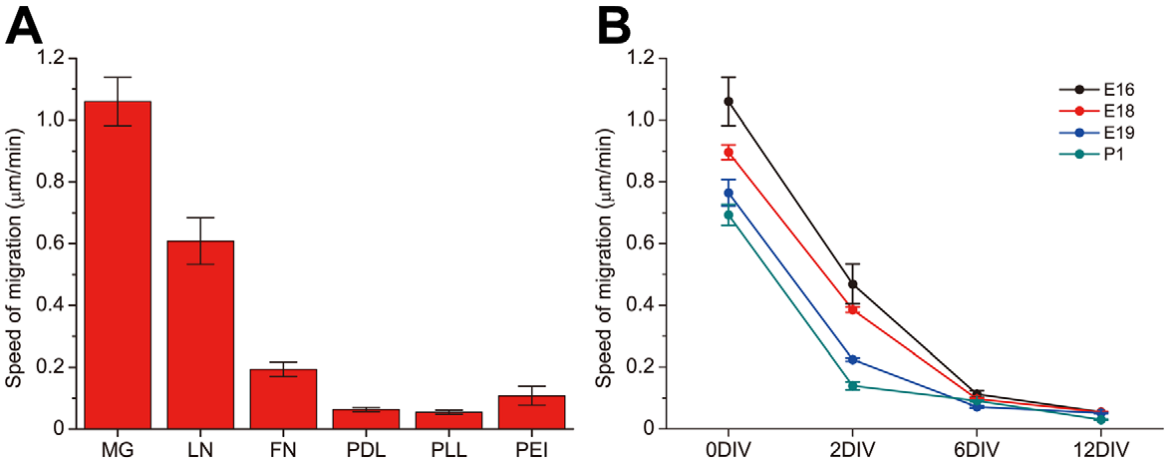
Surface coating and developmental stage modulates the speed of neuronal migration on gold substrate. A, The speed of migration of neurons from E16 rat pups at 0 DIV, cultured on MG coated gold surfaces is significantly higher than those on other surfaces. (MG: n = 24, LN: n = 20, FN: n = 20, PDL: n = 20, PLL: n = 18, PEI: n = 8. P<0.01. One way ANOVA followed by Tukey's *post hoc* test, see Figure S1 for details). The speed of migration is determined by time-lapse imaging at a rate of 10 seconds per frame and calculating the speed by dividing the displacement by the time interval. Note that the long term displacement is zero for any surface coating other than MG and LN (refer to the **Materials and Methods** section for details). B, The speed of migration of neurons at different developmental stages. The speed of migration decreases against the age of the donor animals and the culture. (E16: n = 5, 30 data points at each time; E18: n = 8, 30 data points at each time; E19: n = 8, 30 data points at each time; P1: n = 8, 30 data points at each time. Spearman's *rho* test was used for monotonicity between speed of migration and age of culture, correlation coefficients are E16: 0.77856, P<0.001; E18: 0.70401, P<0.001; E19: 0.70456, P<0.001; P1: 0.80623, P<0.001. Mann- Kendall's *tau* test for monotonicity between speed of migration and age of donor animal (P1 treated as E23), correlation coefficients are 0DIV: 0.40882, P<0.001; 2DIV: 0.30561, P<0.001; 6DIV: 0.22818, P<0.001; 12DIV: 0.31062, P<0.001.) Error bars denote S.E.M.

We found that the speed of migration depends on the surface treatment and donor animal age (Figure 1, see **Materials and Methods** for details on measurement of migration). Neurons migrate very slowly on polymeric surfaces such as poly-D-lysine (PDL), poly-L-lysine (PLL), polyethyleneimine (PEI), as well as the extracellular matrix protein fibronectin (FN) (Figure 1A, Table 1). While they do migrate on laminin (LN); the speed of migration is significantly lower than those on MG-treated surfaces (Figure S1). For example, the speed of migration on MG-coated surfaces (1.06±0.08 µm/min, n = 24) is significantly higher than that on PDL (0.05±0.02 µm/min, n = 8, P<0.0001, One-way ANOVA followed by Tukey's *post hoc* test, Figure 1A). As the age of the donor animal or cell culture increases, the speed of migration decreases (Figure 1B). The speed of migration of neurons from E16 rat pups on Matrigel is the highest and relatively stable (with the lowest coefficient of variation, Table 1), and was used throughout the paper.

**Table 1:** The speed of neuronal migration on MG coated gold surfaces is the highest and the variance lowest.

	MG	LN	FN	PDL	PLL	PEI
*n*	24	20	20	20	18	8
mean (µm/min)	1.05973	0.60849	0.19318	0.06329	0.05485	0.10814
S.E.M.	0.07813	0.07557	0.02355	0.00662	0.00676	0.03086
C.V.	0.36117	0.55539	0.54523	0.46758	0.5233	0.80705

We plated neurons onto patterned SAMs[17], [18], [19], [20], [21] throughout the paper (refer to the **Materials and Methods** for details) to apply geometric constraints on neuronal culture, in order to control the geometry of the neuronal circuit[22], [23], [24], [25], [26]. Briefly, patterns promoting neuron adhesion were formed through micro-contact printing (µCP) with alkanethiols terminated with methyl groups (-CH_3_) on gold substrates evaporated on glass cover slips. The gold substrates were immersed in poly-ethylene glycol (-EG_6_) terminated alkanethiols to make the rest of the surfaces anti-fouling. The constraint could assume arbitrary geometry by design to limit the number of neurons present on an island. We treated the surfaces with MG. Neuronal migration is spatially constrained within the restrictions. In combination with time-lapse imaging, this method facilitates spatiotemporal analysis of the collective migratory behavior of all neuronal cells that constitute an independent neuronal circuit.

### Correlated neurite fasciculation and soma migration

**Figure 2:**
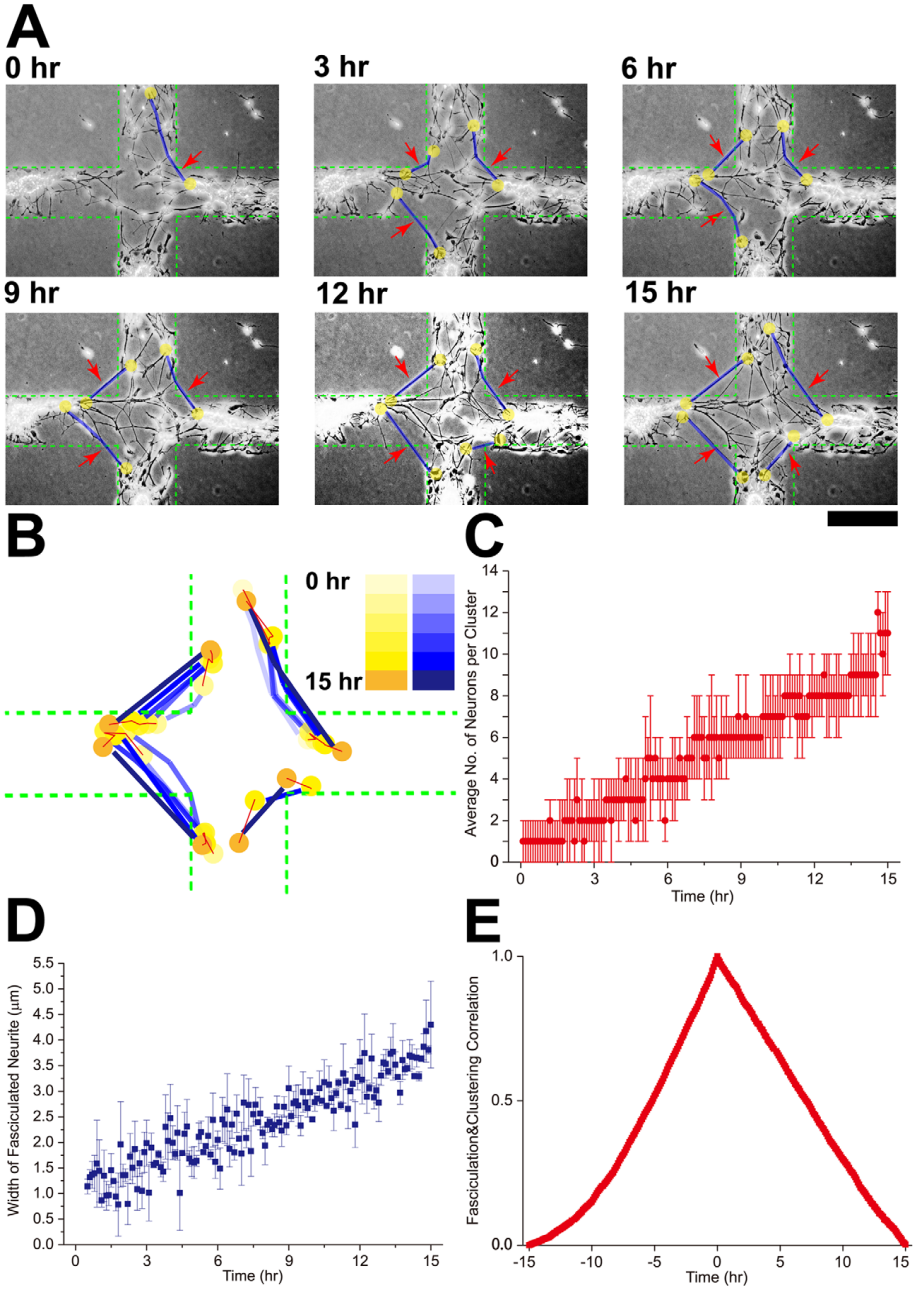
Correlated neurite fasciculation and somata migration. A, Time lapse imaging showing the emergence and evolution of fasciculated neurites (highlighted by light blue lines) accompanied by somata (highlighted by yellow balls) migration over 15 hrs at 3DIV. The geometric constraint was specifically designed so that neuronal somata could only migrate along the area of the cross-shaped geometric constraint (confined to the green dashes) while neurites connecting clusters are not limited by the geometric constraint. B, Color-coded superimposed silhouettes of fasciculated neurites and somata clusters from panel A illustrates the relationship between fasciculation and clustering. A series of blue colors represent the neurites while yellow shades represent the clusters, the depth of color grows as time elapses. Red solid lines represent the migratory trajectory of the cluster and green dashes show the edges of the geometric constraint. C, The average number of neurons within a cluster grows (from 1±1 to 11±2 during the 15 hrs time window, n = 5, 20 data points for each time point) as time elapses, indicating time-evolved recruitment of neurons. D, The width of the fasciculated neurites grows (from 0.91047±0.1003 µm to 4.30173±0.84857 µm, n = 5, 20 data points for each time point) over time. E, Correlation between the number of neurons (Figure 2C) and the width of neurites (Figure 2D). The horizontal axis represents the temporal shift between the two variables, and the highest value is achieved with no time shift, meaning these two events are temporally correlated. Scale bar: 200 µm.

Migration of individual neurons is gradually coupled into collective migration as neurons adhere to each other through neurite fasciculation. At first, soon after adhesion (0 DIV), neurons are unconnected and neuronal migration is disordered. Accompanying the development of the neurites, adjacent neurites come into contact with each other, through migration and neurite development, and depart again. After a period of dynamic interactions, some of the neurites adhere to each other through cell adhesion molecules[27], leading to neurite fasciculation. Neurite fasciculation at this stage is mechanically vulnerable and dynamic, and its impact on neuronal migration is still not obvious. Fasciculated neurites gradually become strengthened and affect neuronal migration[28]. We used a cross-shaped geometric constraint (Figure 2A) to illustrate the relationship between neurite fasciculation and somata migration at 3 DIV. The somata are confined to migrate within the two branches of the cross, while neurites become fasciculated and not restricted by the geometric constraint (note that neurites do not adhere on areas outside of the geometry). Gradually, the formation of fasciculated neurites (Fig. 2A, red arrows) accompanies the formation of cell assemblies (yellow dots) on the two branches of the cross. This correlation is more clearly seen in the superimposed silhouettes in Figure 2B. We further quantified the number of neurons within each cluster (1±1 to 11±2 during the 15 hrs time window, n = 5, 20 data points for each time point, Figure 2C) and the width of fasciculated neurites (0.91047±0.1003 µm to 4.30173±0.84857 µm, n = 5, 20 data points for each time point, Figure 2D), both showing a trend of increase with respect to time. Increasing number of neurons in each cluster corresponds to continuous clustering while increasing width of neurites indicates continuous fasciculation. We found that these two processes are temporally correlated (Figure 2E).

Collective migration on two-dimensional boundless surfaces is self-organized with somata clustering and neurite fasciculation. Similar results have been reported [29], [30]. In this paper, we focus on how geometric boundary conditions affect the dynamics of collective neuronal migration, and therefore the self-organizing circuit assembly.

### Spatiotemporally coordinated collective neuronal migration on neuronal circuits with defined geometric boundaries

We studied the effect of the boundary condition on the dynamics of collective neuronal migration through SAMs-based geometric constraints.

**Figure 3:**
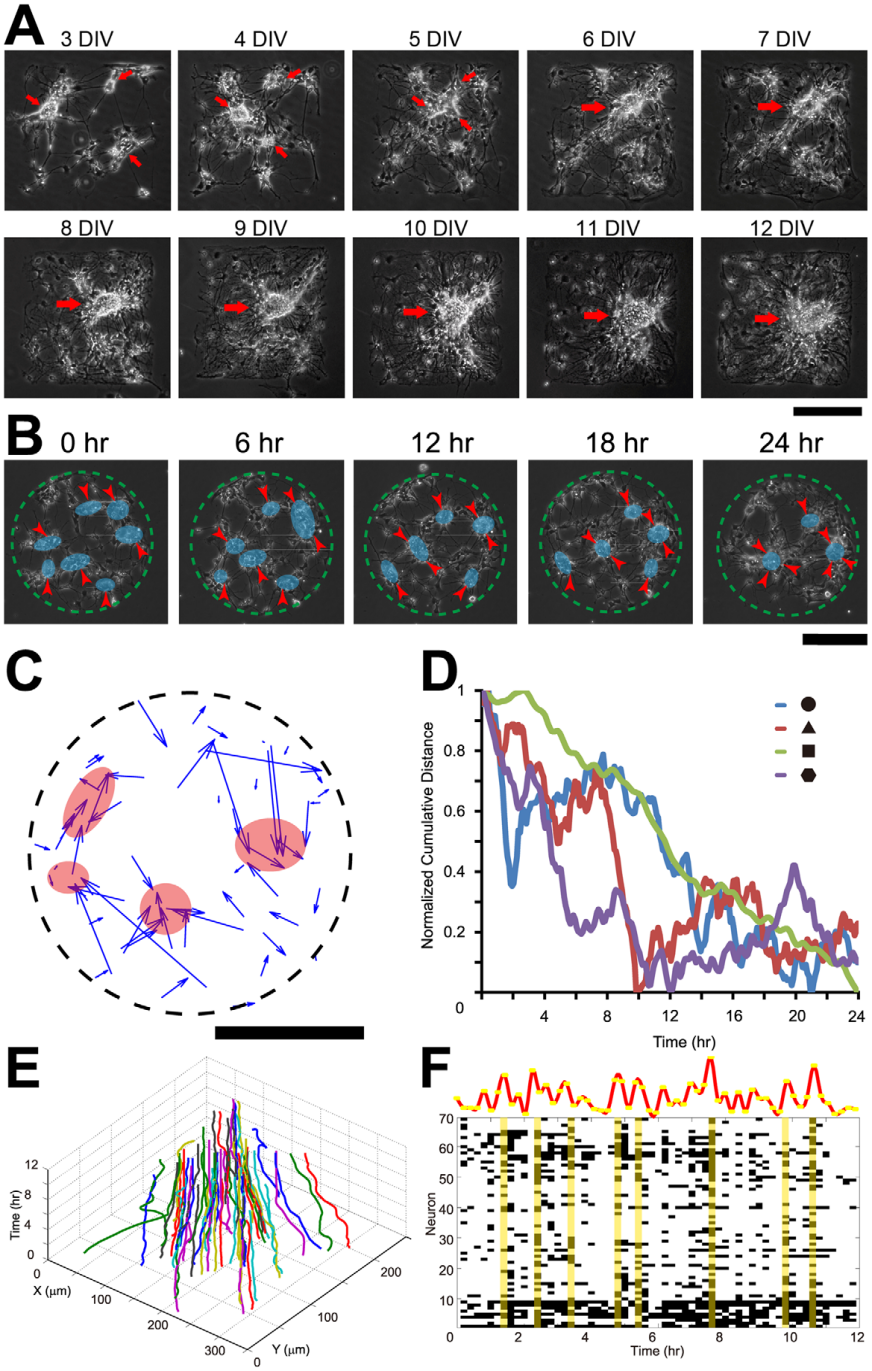
Collective dynamics of neuronal migration within geometric constraints is spatiotemporally coordinated. A, Time-lapse imaging of a circuit maintained over 12 DIV. The red arrows indicate clusters. As shown here, the clusters gradually come together and finally merge into a mono-cluster. B, Collective neuronal migration over 24 hrs on a circular SAMs geometric constraint (depicted by green dashes) at 2 DIV. Transparent blue shades highlight the clustered somata that are coordinated in migration, red arrows designate the orientation of the migrating clusters. As time elapses, smaller clusters merge into larger ones and gradually approach the geometric center of the geometric constraint. C, The behavior of migration of all 36 neurons (at a lower density) on a circle (black dashes) over 24 hrs at 2 DIV at an interval of 5 min. The blue arrow begins from the original position of the cells at 0 hr, and ends at the final location at 24 hr. Neurons that were evenly distributed throughout the geometric constraint at the beginning finally migrate into a few clusters (transparent pink shades) in the end. D, Cumulative distance curves of all neurons on geometric constraints of different shapes (circular, triangular, square and hexagonal) over 24 hrs at 2 DIV at an interval of 5 min (see **Materials and Methods** for details on the definition of the curve). E, Collective pattern of neuronal migration on a square-shaped constraint over 12 hrs at 4 DIV at an interval of 5 min. The X-Y axes indicate the spatial locations of all neurons at a specific time, which is represented by the vertical axis. A trace of a particular color corresponds to one neuron. F, The migratory speed of neurons on a square-shaped constraint over 12 hrs at 4 DIV at an interval of 5 min as in panel E is temporally coordinated (P<0.001, Pearson's χ^2^ test, compared with the temporally shuffled random data, see **Materials and Methods** for details on data analysis). Each black block represents the speed of the specific neuron (indexed by the vertical axis of the block) at a specific time when the speed of migration (indexed by the horizontal axis of the block) is high (above 30%). The vertical axis is the serial number of neurons. The yellow shades highlight time points when most of neurons are migrating at a higher speed. The red curve above is the histogram of the raster plot, showing the number of neurons migrating at a higher speed at each time. The yellow dots represent the number of neurons migrating at high speed at each time point. Scale bars: 200 µm.

Figure 3A shows a series of observations on a square-shaped geometric constraint over 12 days. We found that small clusters (Figure 3A, red arrows) finally merge into each other, forming large clusters. We found that DIV 2-3 is a critical time window in neurite fasciculation and cluster formation. Before this time point, collective neuronal migration is largely uncorrelated (Figure S2), although neurons show a trend of slowing down in migration. Since the critical time point, collective neuronal migration undergoes drastic change and become correlated. We studied neuronal migration at this critical stage within a circle (Figure 3B and 3C). Figure 3B shows the migration of clusters over 24 hrs at 2 DIV. Each blue shaded area covers a cluster while red arrowheads show the migration direction of the clusters. Several small clusters gradually migrate into each other and finally merge into fewer numbers of clusters. Under a specific geometric scale, the clusters merge into a single one, as will be fully discussed in the next section. We analyzed a network on which the cells were plated at a very low density so that each cell was clearly discernable (Figure 3C). We traced the migration of all neurons in the circuit. We use an arrow to indicate the general direction and distance of the migratory pathway of each neuron, where each arrow begins from the original location of a neuron and pointing to the final location. While the cells were evenly distributed throughout the geometric constraints at the beginning, they finally become clustered into a few spots highlighted by pink shades. Clustering takes place consistently, regardless of their initial density. To quantify the trend of migration of all neurons in a network, we calculated the cumulative distance of all neurons of the network. The cumulative distance is the summary of all distances between each pair of neurons (refer to **Materials and Methods** for description). We calculated the cumulative distance on geometries of various shapes. As the normalized cumulative distance curves show in Figure 3D, the cumulative distance generally decreases over 24 hrs regardless of the shapes of the geometric constraints.

We found that the migration of a specific neuron depends on its spatial location within the geometric constraint. Figure 3E shows the three-dimensional (3D) plot of collective migratory trajectory. This image is generated by serially stacking the position of all neurons in a network at different time points into a 3D plot. The horizontal axis corresponds to the position of a specific neuron, the vertical axis indicates specific time points. The starting time of image capture is set to 0 and the value on the vertical axis increases over time. The migration of all neurons tends to be oriented towards the center (Fig. 3E), indicating spatial coordination. Temporally, the speed of neuronal migration is synchronized in that neurons tend to speed up and slow down during migration at the same pace, indicating that the temporal dynamics of collective neuronal migration is also coordinated (Figure 3F, P<0.001, Pearson's χ^2^ test, compared with the temporally shuffled random data, see **Materials and Methods** for description of the statistical analysis). Taken together, we demonstrate that collective neuronal migration within geometric constraint is spatiotemporally coordinated.

We also studied the heterogeneity of collective migration, by performing covariance analysis of collective neuronal migration (Figure S3).We classified the neurons into two populations according to their covariance coefficients (k-means clustering, Figure S3B), and found two types of distinct dynamics of neuronal migration. Figure S3B is the planar projection of Figure 3E. One population of neurons (blue) generally locate in the peripheral zone, spanning across significantly longer ranges (Figure S3C, P<0.001, Kolmogorov-Smirnov test) and assuming higher migratory speed; while the other population of the neurons (red) tend to be located in the center, showing a centripetal pattern of migration.

### Emergence and centrality of mono-cluster is self-organized and sensitive to geometry

**Figure 4:**
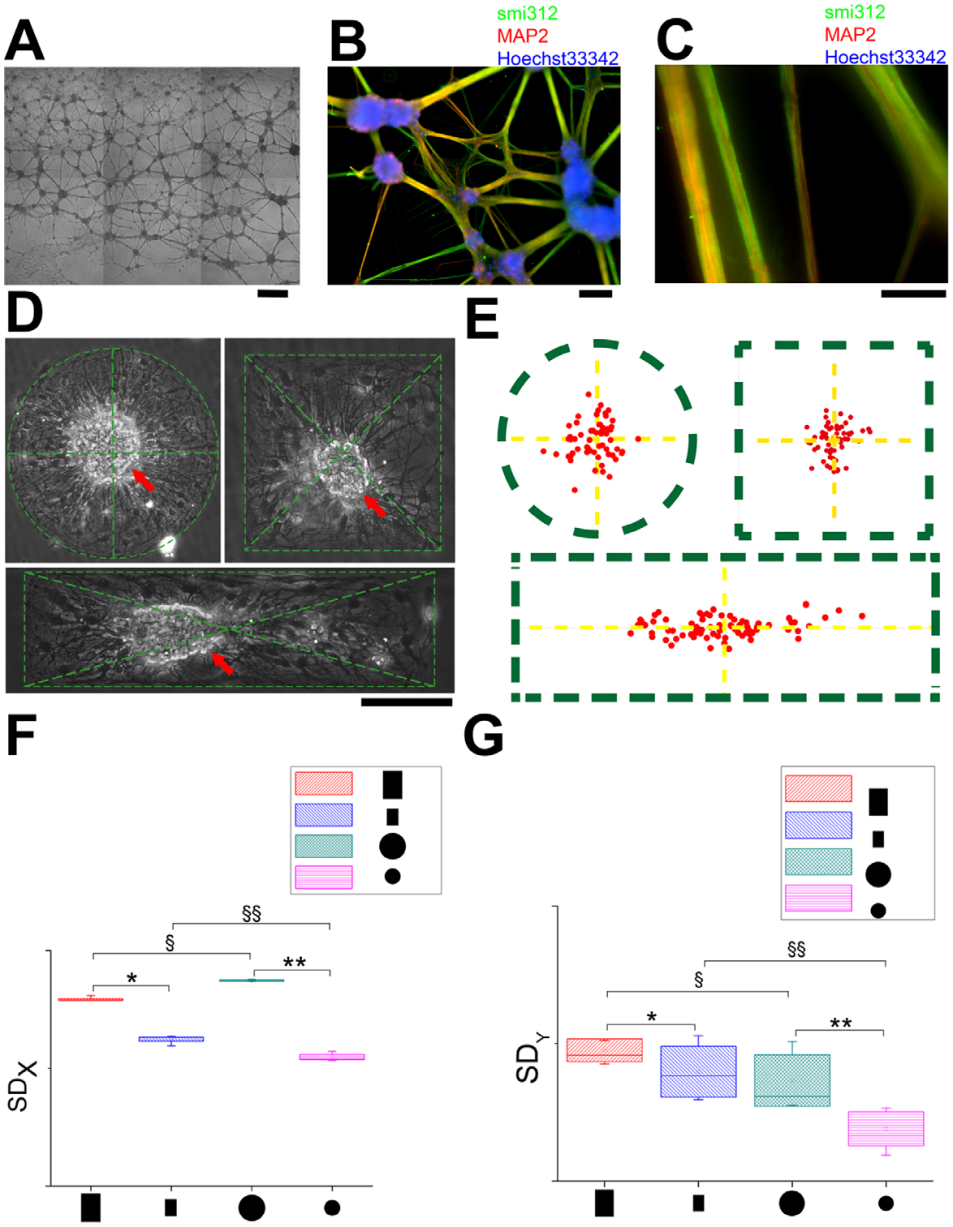
Emergence and centrality of mono-cluster on critically-scaled geometric constraints. A, Phase-contrast photomicrograph of live neurons cultured without geometric constraints at 21 DIV. Individually plated neurons have assembled into randomly located clusters joined through fasciculated neurites. B, Immunocytochemistry showing highly clustered neuronal networks connected by thick and tight fasciculated neurites in panel A. Smi312 is the specific marker of axons, while MAP2 is the specific marker of dendrites. Nuclei are counterstained with Hoechst33342. C, Fasciculated neurites in panel B under higher magnification, the neurites are composed of both axons and dendrites. D, Phase-contrast image of circular, square and rectangular geometric constraints and the deviation of the cluster from the geometric center. Clusters tend to be located in the center on isotropic geometric constraints but retain a degree of freedom on anisotropic geometric constraints like the rectangular geometric constraints. E, Locations of the centers of the clusters on circular, square and rectangular geometric constraints (same as those in panel D) as depicted by the green dashes. The mean radial displacements of the centers of the clusters from the geometric centers are 8±4 µm (circle) 9±6 µm (square) and 32±9 µm (rectangle). The centers are located approximately on the geometric center of the geometry on circular and square constraints and along the central axis parallel to the longer edge on rectangular constraints. (Circular geometric constraint: n = 5, 60 data points; square geometric constraint: n = 5, 60 data points; rectangular geometric constraint: n = 5, 80 data points. The figure is of the same scale as panel D.) F - G, Centrality analysis of circular and rectangular arrays (see Figure S4 for the original image and data extraction procedure). The mean displacements for different shapes are: large rectangles, X = 53±12 µm, Y = 80±34 µm; small rectangle, X = 31±8 µm, Y = 58±29 µm; large circle, X = 65±23 µm, Y = 69±25 µm; small circle, X = 41±11 µm, Y = 38±9 µm. The figures show the box chart of standard deviation of X (upper) and Y (lower) positions among different geometric constraints (SD_X_ and SD_Y_ represents standard deviation in x and y directions, respectively. SD_X_: *, n = 8, P<0.001. **, n = 8, P<0.001. §, n = 8, P<0.001. §§,n = 8, P<0.001. SD_Y_ : *, n = 8, 0.01<P<0.1. **, n = 8, P<0.001. §, n = 8, 0.01<P<0.1. §§,n = 8, P<0.001. One-way ANOVA followed by Tukey-Kramer's *post-hoc* multiple comparisons test). Scale bars: A, 300 µm; B, 100 µm; C, 50 µm; D: 200 µm.

When this clustering process takes place on geometric constraints, the final location of the cluster and the trajectory of migration depend on the shape of the geometric constraints. The clusters generally locate in the geometric center on isotropic restrictions. On anisotropic restrictions, the locations of the clusters tend to be in the central region of the geometric constraint, albeit with a relatively large range of distribution (Figure 4D). This is entirely different from the situation where neurons grow in unrestrained culture. On un-constrained gold substrates (25×25 mm^2^) that could be considered an infinitely large two-dimensional plane with respect to the scale of a single neuron (approximately 6 orders of magnitude larger), the networks are dominated by randomly located clusters (Figure 4A - C). We plotted the locations of the mono-clusters on circular, square and rectangular restrictions (Figure 4E) and analyzed their mean displacements with respect to their geometric centers respectively (8±4 µm (circle, n = 5, 60 data points); 9±6 µm (square, n = 5, 60 data points) and 32±9 µm (rectangle, n = 5, 80 data points). The locations of the clusters fall around the geometric centers of the circuit. They form a band along the longer axis of the rectangle.

In order to verify the robustness of the mono-cluster and centrality property, we extracted the location of the clusters on an array of circular and rectangular restrictions of the same area (see Figure S4 for details on the experimental setup). In all geometries we analyzed, centralized mono-clusters were consistently observed. Figure 4F and 4G are box plots of standard deviation of X and Y values respectively (The mean displacements for different shapes are: large rectangles, X = 53±12 µm, Y = 80±34 µm; small rectangle, X = 31±8 µm, Y = 58±29 µm; large circle, X = 65±23 µm, Y = 69±25 µm; small circle, X = 41±11 µm, Y = 38±9 µm). As shown here, the fluctuations along Y on rectangles are always larger than that on circles (§, n = 8, 0.01<P<0.1. §§,n = 8, P<0.001. One-way ANOVA followed by Tukey-Kramer's post-hoc multiple comparisons test). Larger geometric constraints also accommodate wider range of fluctuations (SD_X_: *, n = 8, P<0.001. **, n = 8, P<0.001. SD_Y_ : *, n = 8, 0.01<P<0.1. **, n = 8, P<0.001. One-way ANOVA followed by Tukey-Kramer's post-hoc multiple comparisons test). By contrast, there is much less fluctuation along the X axis than along the Y axis on rectangular shape (large rectangles: P<0.001, small rectangles: P<0.001, Kolmogorov-Smirnov test).

**Figure 5:**
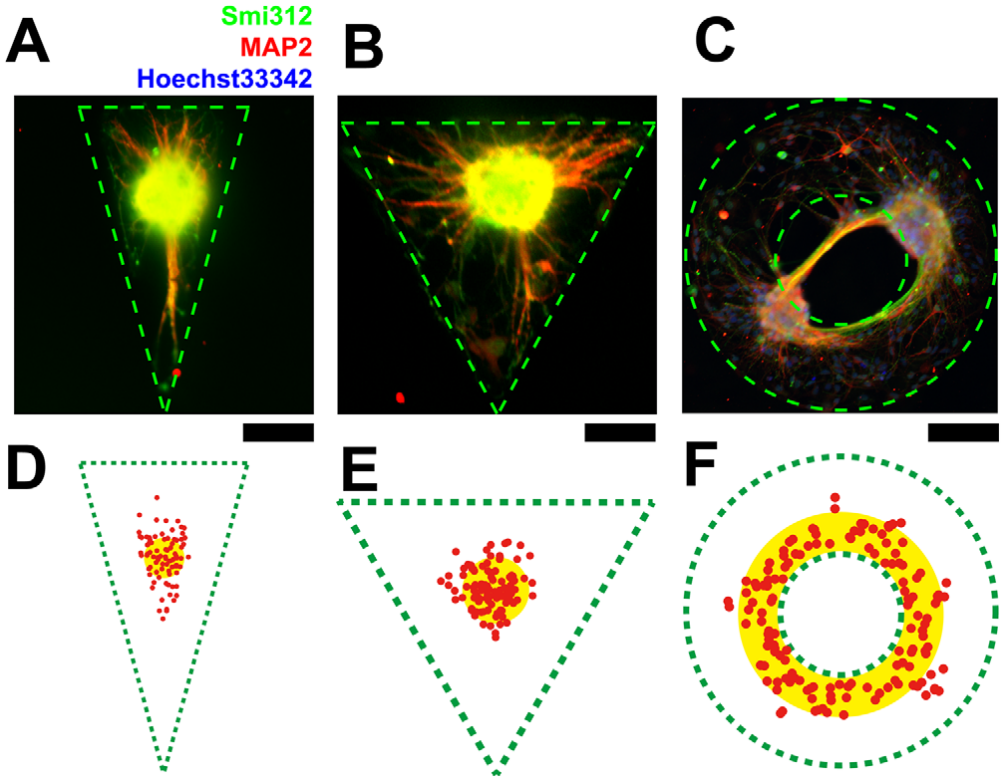
Geometric regulation of clustering location. A - C, Immunocytochemistry with Smi312 and MAP2 showing cluster location within polarized geometric constraints (A: isosceles triangle, B: equilateral triangle, C: concentric ring). D - F, Locations of the centers of the clusters (red dots) within isosceles triangles, equilateral triangles and concentric rings (corresponding to those in panel A - C, with the same scale) as depicted by the green dashes. The mean radial displacements of the centers of the clusters from the geometric centers are 12±6 µm (isosceles triangle) and 11±7 µm (equilateral triangle). The clusters on the concentric rings are located around the inner radius. The clusters are located around the geometric center (yellow shades) of the shapes (isosceles triangle: n = 5, 90 data points; equilateral triangle: n = 6, 90 data points; concentric ring: n = 8, 120 data points). Scale bars: 200 µm.

The exact location of the cluster depends on the geometry of the restriction through mechanical interaction between constituent subunits including cells and sub-clusters. By assuming the pair-wise mechanical interactions between sub-clusters to be independent of their distances, and the distribution of the sub-clusters within the geometry to be statistically homogeneous, we theoretically estimated the direction of the resultant force of all elements in the circuit acting on arbitrary sub-cluster to be pointing towards the centroid of the geometry of the circuit (refer to **Materials and Methods** for details). Therefore, the coordinated migration is centripetal. Consequently, the mono-cluster, where it emerges, is located on the geometric center of the constraint. This is in agreement with our experimental observations (Figure 4E, Figure 5A and 5B). When the geometric centroid lies outside of the constraint (Figure 5C), the cluster tend to locate as close to the centroid as possible within the constraint.

**Figure 6:**
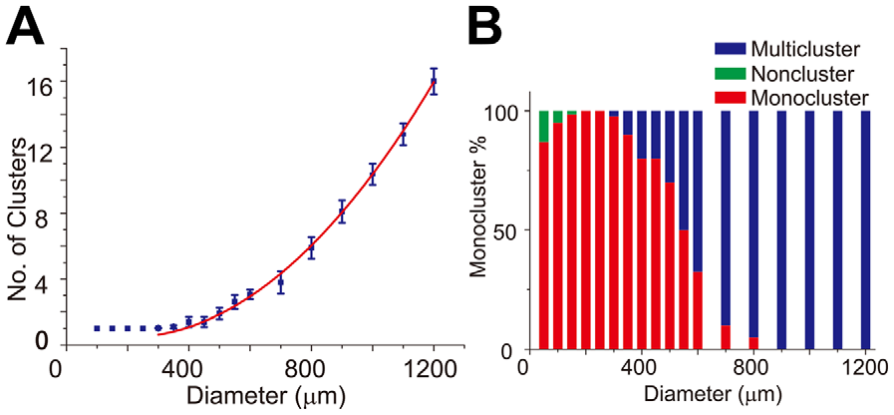
The emergence and centrality of mono-cluster is dependent on the size of geometric constraints. A, The relationship between diameter of the geometric constraint and the number of clusters, red curve is the power law fit (exponent = 2, R = 0.9987, blue error bars denote S.E.M. n = 5, 15–20 data points for each size of geometric constraint.). See Figure S6 for the original data points. B, The percentage (probability) of the appearance of mono-clusters (red) varies with the diameter of the geometric constraint. The highest probability occurs when the diameter of the circular geometric constraint is either 250 or 300 µm. Note that for small geometric constraints there is sometimes no clusters (green) at all, while for large geometric constraints there are multiple clusters (blue). The same set of data as Figure 6A is used.

In addition to shape, the emergence of mono-cluster its location depends on the scale of the network. Using circular-shaped constraints with gradually increasing diameters, we found that mono-cluster has the largest probability to form under 600 µm (Figure 6A, Figure S6A). We performed a linear regression to the cluster data and found a good fit between the diameters of the geometric constraint and the number of clusters (R = 0.9992). In addition, the occurrence of mono-cluster also depends on the diameters of the geometric constraints. On networks smaller than 200 µm, clusters do not always appear, while there might be more than one cluster on larger networks (Figure 6B). Finally, we performed relative neighborhood density[31] analysis on networks of different size (Figure S6B). Quantitative analysis indicates that cells are highly clustered on small networks where neurons are clustered at the center. On larger networks, the curve has a heavier tail, indicating a higher proportion of neurons that are not fully clustered. On free cultures such as those in Figure 4A (25000 µm), the curve has a very mild slope.

In an effort to test the generality of the critical scale, we designed a series of complex networks of identical overall area but composed of decreasing number of units of increasing areas (Figure S5A - C). As shown in the immunofluorescence photomicrographs (Figure S5D and S5G), decreasing the size of the units of the geometric constraints increases the orderliness of the cluster number and location. The number of clusters after long term culture remains a single one on smaller constraints but begins to increase above a threshold range. The threshold appears somewhere between 400 µm and 600 µm, similar to the simple scenario as discussed above (Figure 6). On networks with subunits above the critical scale, the basic network structure is similar to network motifs on unconfined surfaces (Figure 4A - C).

### Cell-type specific pattern of neuronal distribution within the network

**Figure 7:**
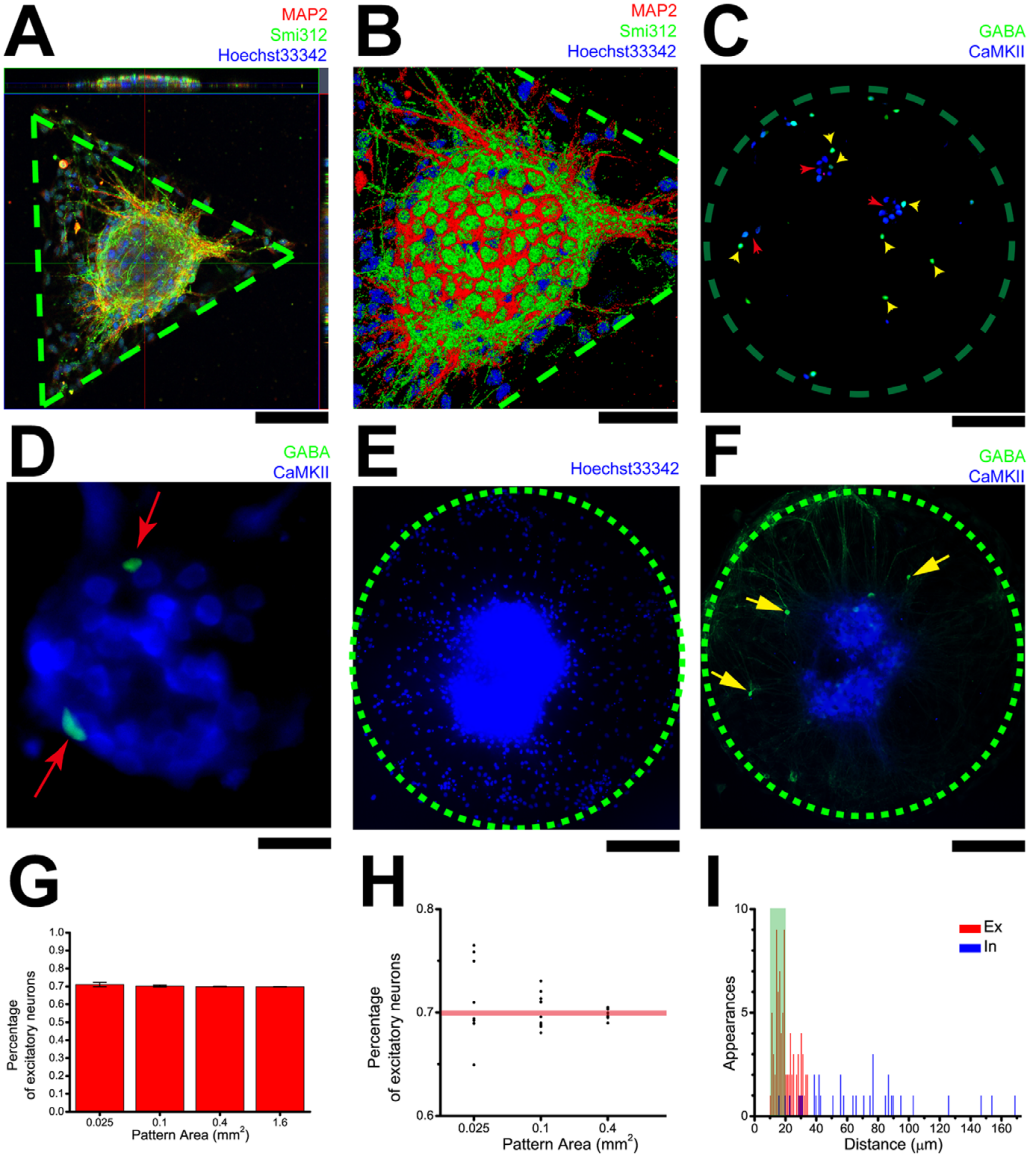
Location and relative number of excitatory and inhibitory neurons within a network. A, A clustered network on a triangle with vertical and horizontal sections. Neuronal dendrites are labeled with MAP2 while axons with Smi312, cell nuclei counterstained with Hoechst 33342. B, Higher magnification of the highlighted cluster in a. Somata are assembled into a ball. C, Immunocytochemistry shows that excitatory neurons tend to cluster more than inhibitory neurons do. Excitatory neurons are labeled with CaMKII while inhibitory neurons are labeled with GABA (refer to the text for details). D, Higher magnification of a cluster. Inhibitory neurons labeled by GABA locate in the periphery of the cluster that is primarily composed of excitatory neurons. E, The location of all cell nuclei within a network. F, The location of excitatory and inhibitory neurons on the same network as E, while most of the neuron somata are clustered in the center of the network, a few somata of inhibitory neurons only are scattered in the periphery of the network. G, The percentage of excitatory neurons in the overall population on geometric constraints of different sizes. Note that there is substantial fluctuation of the total number of neurons among geometric constraints. As the area grows, the ratio gradually approaches unrestrained cultures with large number of cells. (n = 5, 10–15 data points for each size of geometric constraint.) H, Scatterogram showing the distribution of the ratio on geometric constraints of various areas. As the area increases, less fluctuation is present in the percentage. The same set of data as panel G is used. I, Histogram showing that inhibitory neurons tend to stay away from the clusters, indicating a heterogeneous nature in neuronal migration and cluster formation. The green shade represents the range where the diameters of neurons generally fall within. (n = 5, 42 excitatory neurons, 21 inhibitory neurons.) Scale bars: A, 200 µm; B, 25 µm; C, 200 µm; D, 50 µm.

We found that the cluster is composed of a high density of neuronal somata (Figure 7A, 7B). As functional neuronal networks are composed of a balanced combination of excitatory and inhibitory neurons, we set out to analyze the cellular types on networks. Using immunocytochemistry against excitatory and inhibitory neuron markers, α-Ca^2+^/Calmodulin dependent protein kinase II (CaMK II), and γ-aminobutyric acid (GABA), respectively (Figure 7C), we found that the ratio of excitatory versus inhibitory neurons is maintained (Figure 7G) regardless of the number of neurons of the circuit as the area of the constraint grows, with 70% of excitatory neurons and 30% of inhibitory neurons, in accordance with previous reports[32], [33]. There are some fluctuations of the exact value, but as the restriction area increases the value converges towards the value on unrestricted culture (Figure 7H). The clusters (in the center of the geometric constraint) are mostly composed of excitatory neurons (Figure 7D) with inhibitory neurons in the periphery. We frequently found inhibitory neurons far away from the cluster, while excitatory neurons are seldom seen outside the cluster. On a fully mono-clustered network (Figure 7E), the cluster is composed of mostly excitatory neurons while a few inhibitory neurons could be observed in the periphery of the geometric constraint far away from the cluster (Figure 7F). We analyzed the distance between arbitrary pair of cells, and sorted the distances into two groups, one group is the distance between a pair of excitatory neurons and the other the distance between an inhibitory neuron and an arbitrary neuron. As the histogram shows (Figure 7I), the excitatory neurons tend to cluster together while inhibitory neurons stay far away from each other. This result is also evident in Figure 7C. Interestingly, the peak of the histogram overlaps with the range of conventional soma diameters (data acquired from neurons in the same culture experiment), indicating that most of the excitatory neurons are clustered.

## Discussion

We demonstrate that neural circuits within critically-scaled geometric constraints exhibit spatiotemporally coordinated pattern of migration (Figure 3) and mono-clustered network structure (Figure 4 and Figure 5) on MG-coated SAM surfaces, in contrast to disordered neuronal migration and circuit structure (Figure 5A - 5C) in the absence of geometric constraints. Besides, neural circuits adapt to various geometric inputs in a circuit-autonomous manner. (Figures 4, 5, 6, Figure S5). Taken together, these results indicate that developing neural circuits are self-organized within geometric constraints. The assembly of neural circuits is regulated by both genetics [34], [35] and neural activity [6], [36]. However, molecular cues and neural activity take effects in the form of spatiotemporal patterns onto neural circuits. Geometric constraints have been found to regulate non-neuronal cell migration *in vitro* [18], [37]. How geometric distribution of molecular cues and neural activity regulates circuit assembly remains an open question[38]. Our *in vitro* study shows geometric control of circuit assembly, which could be a developmental strategy readily implementable *in vivo*.

We found that neuronal migration and neurite fasciculation are correlated (Figure 2E). Fasciculated neurites linking clustered somata tend to be straight (Figure 2, Figure 4A and 4B), indicating that the neurites are in tension. The tension could be caused by traction force reciprocally exerted by two clusters onto each other, and the traction force mediated through these fasciculated neurites would in turn affect neuronal migration. When most of the neurons are connected through these fasciculated neurites in tension, the circuit would be a mechanically coupled entity (Figure 2, Figure 4A, 4B, 4C), and neuronal migration within the circuit become globally coordinated.

We built a working model based on the experimental observations (Figure S7). On an infinitely large network populated with neurons connected through neurites under tension, the network would be stable if the neurons are stationary and neurites under balanced tension force. However, the cells are migrating and undergoing development, leading to transient fluctuations in tension force, thereby breaking the symmetry of mechanical equilibrium in the neurites, and the cell exerting stronger force would drag adjacent neurons closer, facilitating adhesion and clustering. This pair-wise process scales up on a network with a large amount of neurons to recruit and multiple fasciculated neurites for each neuron, causing the formation of hierarchically larger clusters (Figure S7B). Accompanying the formation of hierarchically larger clusters, the migration of neurons in a network might be coordinated into the migration of hierarchically larger clusters, via mechanical interactions between the clusters linked by fasciculated neurites. Based on this working model, we built a simple theoretical model (see **Materials and Methods**) and predict that mono-clusters tend to locate on the centroid of the geometric constraint (Figure S7D), which is in agreement with experimental results (Figure 4, 5). However, our theoretical model assumes that the force between sub-clusters is independent of the distance, which would be violated over longer distances (Figure S7C), and above a critical scale, where reliable propagation and coordination fails, leaving local clusters un-centralized (Figure 4A and 4B). The application of geometric constraint defines the range of mechanical coupling, and therefore different patterns of collective migration and circuit assembly (Figures 4, 5, 6, Figure S5). Similar results of the dependence of emergent property on a critical scale has been demonstrated in other coupled systems such as the microbial oscillators[11] in which microfluidic channel played a similar role as SAMs-based geometric constraints here. While mechanical coupling has long been hypothesized to influence circuit assembly[39], and several groups have addressed this issue through experimental and theoretical approaches[40], [41], [42], [43], [44], [45], [46], [47], our work proposes a potential role played by mechanical tension in coordinating collective neuronal migration within geometric constraint, thereby regulating circuit assembly.

Clustering permeates neural networks *in vivo* and *in vitro* at synapses[48] and neurites[49]. Soma clustering exists in a variety of central nervous systems, in which somata are grouped together into grey matter while neurites white matter. This segregation may be evolutionarily advantageous due to economy[50]. Our results indicate that circuit-autonomous self-organization in response to geometric control is sufficient for the realization of similar structures through neuronal migration *in vitro* (Figure 7A and 7B). Our discovery of self-organized formation of clusters under specific geometric constraints possibly indicates that clustered somata distribution is the most economical pattern of wiring[51]. Self-organization has been found in various physical[52] and biological subjects[10]. It has been implicated in the morphogenesis of tissues ranging from hair[9], pancreatic islets[53], optic cup[8] to the brain[49], [54], [55]. As geometric constraints have been shown to affect circuit function[56], the interaction between geometry and wiring, as a case study of formation and function, could be a general strategy for wiring optimization during evolution[57]. Besides, neuronal morphology with elongated neurites might be a structural basis for wiring optimization[39].

We found cell-type specific clustering of excitatory and inhibitory neurons (Figure 7C - 7I). This is possibly correlated with different patterns of neuronal migration (Figure S3). In fact, excitatory neurons migrate along a trajectory different from inhibitory neurons in the brain. As excitatory-inhibitory interaction and balance is critical in neural physiology and pathophysiology[58], [59], cell-type specific clustering might be the structural basis of neural computation within local microcircuits[60], [61].

Precise engineering of the circuit map is a prerequisite for constructing functional neural circuits for neural prosthetics, regenerative medicine and fundamental research[62], [63]. Controlling the relative position of the neuronal somata within the networks is a key step in neural circuit engineering. The elucidation of the presence of critical scale is important for engineering two-dimensional neural circuits, and shed light on the engineering of three-dimensional neuronal networks through scaffold with critically-scaled geometric features[64]. The model system established here could be used for establishing disease models of neurological disorders[65] and for quantitative analysis of complex systems[66], [67].

## Materials and Methods

### Microfabrication and surface chemistry

We employed standard protocols of soft lithography for fabricating the stamps for micro-contact printing. Briefly, masks were designed with AutoCAD (Autodesk) and generated with high-resolution printing, SU-8 (SU-8 2025 and 2100, MicroChem) mold were made through standard photolithography with a mask aligner (MJB4, Suss MicroTec), soft lithography[20], [68] of polydimethylsiloxane (PDMS, Dow Corning) was performed to obtain the elastic stamps for µCP.

SAMs were formed according to standard protocol with alkanethiols on gold substrates[17], [18], [69], [70], [71]. Gold substrates were prepared by evaporating a layer of 8 nm-thick titanium followed by 40 nm of gold with a vacuum electron-beam evaporator (Edward Auto 500) on acid-treated glass cover slips. Alkanethiols terminated with methyl group -CH_3_ (HS (CH_2_)_15_CH_3_, Sigma) were used for creating patterns to promote cell adhesion through µCP. Patterned gold substrates were immersed in poly-ethylene glycol (EG) -EG_6_ terminated thiols (HS(CH_2_)_11_(OCH_2_OCH_2_)_3_OH(C_11_EG_3_), Sigma) for 2 hrs at room temperature to make the rest of the surfaces anti-fouling.

Micro-patterned surfaces were coated with poly-D-lysine (molecular weight 70,000∼150,000, 50 µg/mL in sterile water, Sigma), poly-L-lysine (molecular weight 70,000∼150,000, 50 µg/mL in sterile water, Sigma), laminin (50 µg/mL in Dulbecco's phosphate buffer saline, D-PBS, R&D Systems), fibronectin (50 µg/mL in D-PBS, BD Biosciences), Matrigel (1∶100 v/v in serum free DMEM, BD Biosciences), incubated at 37°C for 2 hrs, followed by rinsing with sterile water (for polymers) or D-PBS (for proteins) and incubated with DMEM/F12 medium (GIBCO) supplemented with 10% of horse serum (GIBCO) at 37°C before plating neurons. All reagents are purchased from Sigma unless otherwise specified.

### Primary culture of rat hippocampal neurons

Methods used for culturing hippocampal neurons follows traditional approaches with adaptions[72], [73]. Neonatal (P0, P1) and embryonic (E16, E18, E19) Sprague-Dawley rats are anesthetized, and hippocampi were isolated and chopped into pieces, treated in 0.25% Trypsin (GIBCO) supplemented with DNase I (Sigma) for 30 min at 37°C, and triturated. Dissociated neurons were seeded onto the surfaces in DEME/F12 medium supplemented by 10% horse serum. After cell adhesion, the surfaces were rinsed gently with DEME/F12 medium twice, and replaced with Neurobasal medium supplemented with 2% B27 and 1% GlutaMax-1 (all from GIBCO) without antibiotics. The medium was replaced by half every four days. All animal experiments were approved by Institutional Animal Care and Use Committee of National Center for Nanoscience and Technology (2009–0214–01).

Each parallel experiment was carried out with new microfabrication and surface chemistry as well as new primary culture. This corresponds to a single count in terms of the number of experiments (designated by *n* in statistics).

### Immunocytochemistry

Cell were rinsed with D-PBS (37°C), fixed in 4% paraformaldehyde for 30 min, permeabilized with 0.3% Triton X-100 for 15 min at ambient temperature. After blocking with 10% goat serum in D-PBS for 1 hr, primary antibodies against Smi-312 (Covance), MAP2 (Millipore), Tuj1 (Sigma), GFAP (Sigma), GABA (Sigma), CaMKII (Invitrogen) were applied, and incubated overnight at 4°C, followed by rinse in D-PBS and visualization with Alexa Fluor 488, 555 or 633 conjugated secondary antibodies (Invitrogen). Cell nuclei were counterstained with Hoechst 33342 (Invitrogen).

### Imaging

Time-lapse imaging was performed on an AF6000 live cell imaging workstation (Leica Microsystems) based on the inverted fluorescence microscope DMI 6000B (Leica Microsystems). For migratory speed analysis as in Figure 1, we used an acquisition interval of 10 seconds between adjacent frames. This particular time interval was determined by analyzing the smallest discernable displacement through the wide-field optical microscope. We found 20 seconds to be the lower bound for migratory speed variation, and 10 seconds was used according to Shannon's sampling theorem.

Fluorescence photomicrographs were taken on a LSM 710 laser scanning confocal microscope (Carl Zeiss).

Enhancement and processing of photomicrographs was done with Photoshop (Adobe).

### Data analysis and statistics

ImageJ (NIH) and Image Pro Plus (Media Cybernetics) were used for semi-automatic quantitative measurements on image data.

Data analysis was performed in Matlab (The MathWorks).

Statistical analysis was performed by R (The R Foundation for Statistical Computing). For groups of two conditions, statistical tests were performed using two-tailed Student's *t* test, Mann-Whitney *U* test or Kolmogorov-Smirnov test. For groups of three or more conditions, one-way ANOVA or Kruskal-Wallis' ANOVA were used, followed by Tukey-Kramer's or Bonferroni's *post hoc* multiple comparisons tests. Polynomial regression was used for curve fitting. Descriptive statistics were presented as mean±S.E.M. unless otherwise noted. All error bars designate S.E.M. Monotonicity was tested via Spearman's *rho* test or Mann- Kendall's *tau* test. K-means clustering was employed for clustering analysis.

Enhancement and processing of vector graphs was done with Illustrator (Adobe).

### Measurement of migration

The measurement of migration is based on position analysis on image series acquired during time-lapse imaging. Specifically, the geometric center of the cell is manually determined (the size of the neuronal soma is relatively small and therefore manual analysis will not introduce much error), and the displacement between adjacent frames was calculated. The speed of migration is defined by the displacement divided by the time interval for image acquisition. The exact time interval depends on the analysis. For the comparison analysis on the speed of migration as in Figure 1, high sampling rate of 10 seconds per frame was employed. For long term monitoring in the rest of the paper, a sampling rate of 5 min per frame was used to reduce the size of the data. Similar methods were used for analyzing acceleration and angle in Figure S2.

### Calculation of normalized cumulative distance

Suppose a network composed of *N* neurons is studied within time duration [*0*, *T*]. The time variant cumulative distance 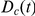 is calculated as the sum of all distances between each pair of neurons:
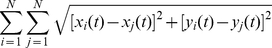
(1.1)while *i*, *j*<*N*, and *0*<*t*<*T*, where 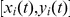 is the location of neuron *i* at time *t*.

The normalized cumulative distance follows:
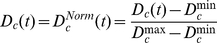
(1.2)in which, 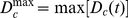
(1.3)

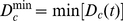
(1.4)Theoretical prediction of cluster location

In a circuit with geometric shape *R* populated with *N* sub-clusters, for arbitrary sub-cluster *i* within *R*, if we designate the tension force mediated through neurite fasciculation between sub-cluster *i* and *j* as 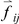, where we assume 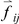 to be independent of the distance between *i* and *j*, such that:
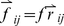
(1.5)in which, *f* is the magnitude of 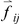 and 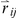 is the unitary vector with the direction from *i* pointing to *j*.

Then for the resultant force of all sub-clusters within *R* acting on sub-cluster *i*:
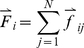
(1.6)we have,
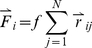
(1.7)


We assume that the N sub-clusters are evenly distributed throughout R, which is likely to be true statistically. Then the term 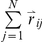 is a vector pointing from *i* to the centroid of the geometric constraint.

Therefore, the direction of the resultant force of all elements in the circuit acting on sub-cluster *i* points toward the centroid of the geometry of the circuit.

### Relative neighborhood density

Relative neighborhood density quantifies the cluster distribution.

For a surface with *N* clusters, the distribution function of cluster *n*(*r*) is a variable that depends on the location of the plane, for arbitrary cluster *i*, we define its specific neighborhood density as:
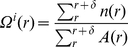
(1.8)


in which *A*(*r*) is the area of an annuli with a distance of r from cluster *i*, with width *δ*,

we then have the relative neighborhood density by averaging over all *N* clusters on the plane:
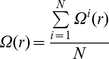
(1.9)


In this paper, the width of the annuli *δ*, which defines the resolution of the analysis, is set to 1.

For an infinitely large two-dimensional plane, suppose the distribution of the neurons is homogenous. Consequently, the distribution function of the neurons *n*(*r*) is a constant independent of *r*. Suppose the density of clusters is *d*, then we have:
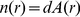
(1.10)The specific neighbor density is:
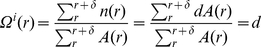
(1.11)the relative neighborhood density is:
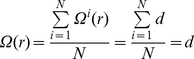
(1.12)which is a constant independent of *r*.

### Temporal synchronization analysis

For Figure 3F, the speed of each neuron is normalized across time. A binarization algorithm is then applied with a threshold of 30% of the highest speed of the neuron. The peak of the temporal bands above threshold is extracted as the time point for synchronization analysis (black block). The time intervals between the time points are then shuffled across cells as the random level. The P values of synchrony level (defined as a probability that the same or higher level of coactivation could occur by chance in 1000 interval-shuffled surrogates[74]) are then compared between the original data and the shuffled random series, and subject to statistical test (Pearson's χ^2^ test). The raster plot is collapsed into the yellow dots, which is then smoothened with a Gaussian kernel to generate the histogram (red curve).

## Supporting Information

Figure S1 **Multiple Comparisons test on the speed of neuronal migration on gold substrates with various surface coatings.** Red error bars denote significant difference (P<0.05), while blue error bars denote non-significant difference. The speed of neuronal migration on MG coating is significantly higher than any other coatings (One-way ANOVA followed by Tukey's post hoc test).
(TIF)

Figure S2 **Neuronal migration in confined space at 0 DIV.** A, The migration trajectories of 135 neurons located on a square-shaped network traced with time-lapse imaging for 12 hours with an interval of 5 minutes between each frame. Each colored line corresponds to an individual neuron. Green dashes depict the geometry of the geometric constraint. B, Phase-contrast image of the first and the last frame, red arrows point to emerging clusters. C - H, Motion analysis of the neurons shown in panel A over 12 hr in terms of velocity, acceleration and angle. C, E, G are superimposed velocity, acceleration and angle of all 135 neurons in the network over time. Huge variations in migration capability between neurons in terms of velocity (C), acceleration (E) and angle (G) can be seen. D, F, H are density plots showing the mean (red box) and S.E.M. (line), with a superimposed blue curve showing the behavior of a randomly chosen sample neuron. A gradual decrease of velocity as time elapses can be seen in panel D (Spearman's correlation coefficient is 0.5332, P<0.0001). Sharp bursts in speed take place randomly. While the mean values of acceleration are largely constant, the S.E.M. demonstrate a gradual narrowing over time (panel F, Spearman's correlation coefficient is 0.4582, P<0.0001). The envelope of angle ebb and flow (panel G), but the S.E.M. gradually decreases (panel H, Spearman's correlation coefficient is 0.3213, P<0.0001). Meanwhile, the behavior of a single neuron is characterized by random fluctuations. Scale bar: 200 µm.
(TIF)

Figure S3 **Heterogeneity of collective migration.** A, Color-coded normalized covariance matrix for the velocities of all cells in Figure 3E. The numbers are the serial numbers of the cells. The color of each dot in the matrix represents the covariance coefficient between the two cells designated by the horizontal and vertical index of the matrix respectively. The color bar relates different colors to the covariance coefficients (between 0 and 1). B, The projection of the three-dimensional figure in Figure 3E onto the horizontal plane. We categorized the cells according to their covariance coefficients (panel A) through k-means clustering, cells with larger covariance coefficients are labeled blue while the rest are labeled red. The two populations of neurons exhibit two modes of migration. Neurons with red-colored trajectories are coordinated by the centripetal movement, while neurons with blue-colored trajectories show edge-philic movement. C, Box chart showing the cumulative distance that the red and blue group of neurons travel (* P<0.001, Kolmogorov-Smirnov test, Central: n = 56, Peripheral: n = 13). Scale bar: 200 µm.
(TIF)

Figure S4 **Data extraction strategy for** **Figure 4F and 4G** **.** The X and Y position of each centralized cluster (shown as a green spot in the array) in the rectangular and circular array of geometric constraints are calculated and converted into matrices. The covariance matrices are then calculated according to the position matrices.
(TIF)

Figure S5 **Critical scale determines network structure.** Clustering affects neuronal connectivity, immunocytochemistry photomicrographs of geometric constraints shown in A - C. D - F are part of the network composed of four units. G - I immunocytochemistry of a part of the network and an individual unit. Tuj1 (green) is the neuronal marker. J, Emergence of mono-cluster is size dependent. The number of clusters on a geometric constraint increases as the sizes of the square-shaped geometric constraints expands as a geometric progression (n = 5, 10 - 15 data points for each size of geometric constraint). Scale bar: D - F, 250 µm; G - I, 100 µm.
(TIF)

Figure S6 A, Original data points for Figure 6A. Note that each red dot could be many overlapping data points, such as those of 50 to 250 µm where mono-clusters are consistently observed. B, Normalized relative neighborhood density of geometric constraints of various sizes. The horizontal axis is shifted rightward to show the sharp peaks at zero. See **Materials and Methods** for details. (n = 3, 2000 to 5000 data points for each size of geometric constraint.)
(TIF)

Figure S7 **Working model for geometric control over coordinated collective migration via mechanical coupling.** A, Fasciculation and tension generation between two adjacent neurons. Contact of adjacent neurites (light blue) facilitates adhesion (deep blue velcro) mediated neurite fasciculation, while the somata (yellow balls) are still subject to active migration. The neurites are then subject to tension force (red arrow), possibly caused by the development of neurite fasciculation. Finally, the fasciculated neurites under tension drag the somata closer. B, Formation of larger clusters on a random network composed of small clusters (dark blue circles) fully connected through fasciculated neurites (light blue lines). The break of symmetry on a neurite will result in directed forces along a neurite, leading to somata migration. C, Size dependent difference in network structure. On very small circuits (dashed circle with smallest diameter), there is no cluster. On circuit above critical scale (dashed circle with largest diameter) there would be multi-clusters. Spatiotemporally coordinated migration and mono-cluster appears when the geometry of the circuit falls in between (dashed circle with medium-sized diameter). The blue balls are stabilized clusters under equilibrium in the absence of geometric constraints. The size of the geometric constraint (green dashes) with respect to the critical scale (red circle) determines the potential number of clusters the circuit would host. D, Geometry regulates cluster location. Filled blue circles represent sub-clusters. Blue lines represent fasciculated neurites between the clusters. Red arrows represent traction forces exerted onto the clusters. Filled yellow circles (and yellow ring in the case of concentric ring) represent final locations of clusters. Green dashed lines are the boundaries of the geometric constraints. The centers of the clusters are located on the geometric centers. When the centripetal movement is blocked by a concentric ring, the cluster forms around the ring.
(TIF)

## References

[1] Ayala R, Shu TZ, Tsai LH (2007). Trekking across the brain: The journey of neuronal migration.. Cell.

[2] Rakic P (2009). Evolution of the neocortex: a perspective from developmental biology.. Nature Reviews Neuroscience.

[3] Kriegstein AR, Noctor SC (2004). Patterns of neuronal migration in the embryonic cortex.. Trends in Neurosciences.

[4] Marin O, Rubenstein JLR (2003). Cell migration in the forebrain.. Annual Review of Neuroscience.

[5] Gupta A, Tsai LH, Wynshaw-Boris A (2002). Life is a journey: A genetic look at neocortical development.. Nature Reviews Genetics.

[6] Spitzer NC (2006). Electrical activity in early neuronal development.. Nature.

[7] Guan CB, Xu HT, Jin M, Yuan XB, Poo MM (2007). Long-range Ca2+ signaling from growth cone to soma mediates reversal of neuronal migration induced by Slit-2.. Cell.

[8] Eiraku M, Takata N, Ishibashi H, Kawada M, Sakakura E (2011). Self-organizing optic-cup morphogenesis in three-dimensional culture.. Nature.

[9] Plikus MV, Baker RE, Chen C-C, Fare C, de la Cruz D (2011). Self-Organizing and Stochastic Behaviors During the Regeneration of Hair Stem Cells.. Science.

[10] Karsenti E (2008). Self-organization in cell biology: a brief history.. Nature Reviews Molecular Cell Biology.

[11] Danino T, Mondragon-Palomino O, Tsimring L, Hasty J (2010). A synchronized quorum of genetic clocks.. Nature.

[12] Baruchi I, Ben-Jacob E (2007). Towards neuro-memory-chip: Imprinting multiple memories in cultured neural networks.. Physical Review E.

[13] Schneidman E, Berry MJ, Segev R, Bialek W (2006). Weak pairwise correlations imply strongly correlated network states in a neural population.. Nature.

[14] Gal A, Eytan D, Wallach A, Sandler M, Schiller J (2010). Dynamics of Excitability over Extended Timescales in Cultured Cortical Neurons.. Journal of Neuroscience.

[15] Bakkum DJ, Chao ZC, Potter SM (2008). Long-Term Activity-Dependent Plasticity of Action Potential Propagation Delay and Amplitude in Cortical Networks.. Plos One.

[16] Voigt T, Opitz T, de Lima AD (2005). Activation of early silent synapses by spontaneous synchronous network activity limits the range of neocortical connections.. Journal of Neuroscience.

[17] Sun Y, Liu YY, Qu WS, Jiang XY (2009). Combining nanosurface chemistry and microfluidics for molecular analysis and cell biology.. Analytica Chimica Acta.

[18] Jiang XY, Bruzewicz DA, Wong AP, Piel M, Whitesides GM (2005). Directing cell migration with asymmetric micropatterns.. Proceedings of the National Academy of Sciences of the United States of America.

[19] Chen CS, Mrksich M, Huang S, Whitesides GM, Ingber DE (1997). Geometric control of cell life and death.. Science.

[20] Whitesides GM, Ostuni E, Takayama S, Jiang XY, Ingber DE (2001). Soft lithography in biology and biochemistry.. Annual Review of Biomedical Engineering.

[21] Dupont S, Morsut L, Aragona M, Enzo E, Giulitti S (2011). Role of YAP/TAZ in mechanotransduction.. Nature.

[22] Lau PM, Bi GQ (2005). Synaptic mechanisms of persistent reverberatory activity in neuronal networks.. Proceedings of the National Academy of Sciences of the United States of America.

[23] Wilson NR, Ty MT, Ingber DE, Sur M, Liu GS (2007). Synaptic reorganization in scaled networks of controlled size.. Journal of Neuroscience.

[24] Feinerman O, Rotem A, Moses E (2008). Reliable neuronal logic devices from patterned hippocampal cultures.. Nature Physics.

[25] Bekkers JM, Stevens CF (1991). Excitatory and Inhibitory Autaptic Currents in Isolated Hippocampal-Neurons Maintained in Cell-Culture.. Proceedings of the National Academy of Sciences of the United States of America.

[26] Kleinfeld D, Kahler KH, Hockberger PE (1988). Controlled Outgrowth of Dissociated Neurons on Patterned Substrates.. Journal of Neuroscience.

[27] Hoffman S, Friedlander DR, Chuong CM, Grumet M, Edelman GM (1986). Differential Contributions of Ng-Cam and N-Cam to Cell-Adhesion in Different Neural Regions.. Journal of Cell Biology.

[28] Rutishauser U, Edelman GM (1980). Effects of Fasciculation on the Outgrowth of Neurites from Spinal Ganglia in Culture.. Journal of Cell Biology.

[29] Segev R, Benveniste M, Shapira Y, Ben-Jacob E (2003). Formation of electrically active clusterized neural networks.. Physical Review Letters.

[30] Sorkin R, Gabay T, Blinder P, Baranes D, Ben-Jacob E (2006). Compact self-wiring in cultured neural networks.. Journal of Neural Engineering.

[31] Condit R, Ashton PS, Baker P, Bunyavejchewin S, Gunatilleke S (2000). Spatial patterns in the distribution of tropical tree species.. Science.

[32] Craig AM, Banker G, Chang WR, McGrath ME, Serpinskaya AS (1996). Clustering of gephyrin at GABAergic but not glutamatergic synapses in cultured rat hippocampal neurons.. Journal of Neuroscience.

[33] Fishell G, Rudy B (2011). Mechanisms of Inhibition within the Telencephalon: “Where the Wild Things Are”.. Annual Review of Neuroscience.

[34] Sanes JR, Yamagata M (2009). Many Paths to Synaptic Specificity.. Annual Review of Cell and Developmental Biology.

[35] Williams ME, de Wit J, Ghosh A (2010). Molecular Mechanisms of Synaptic Specificity in Developing Neural Circuits.. Neuron.

[36] Katz LC, Shatz CJ (1996). Synaptic activity and the construction of cortical circuits.. Science.

[37] Mahmud G, Campbell CJ, Bishop KJM, Komarova YA, Chaga O (2009). Directing cell motions on micropatterned ratchets.. Nature Physics.

[38] Laughlin SB, Sejnowski TJ (2003). Communication in neuronal networks.. Science.

[39] VanEssen DC (1997). A tension-based theory of morphogenesis and compact wiring in the central nervous system.. Nature.

[40] Ayali A (2010). The function of mechanical tension in neuronal and network development.. Integrative Biology.

[41] Ayali A, Anava S, Greenbaum A, Ben Jacob E, Hanein Y (2009). The Regulative Role of Neurite Mechanical Tension in Network Development.. Biophysical Journal.

[42] Hanein Y, Tadmor O, Anava S, Ayali A (2011). Neuronal soma migration is determined by neurite tension.. Neuroscience.

[43] Rajagopalan J, Tofangchi A, A Saif MT (2010). Drosophila Neurons Actively Regulate Axonal Tension In Vivo.. Biophysical Journal.

[44] Saif T, Siechen S, Yang SY, Chiba A (2009). Mechanical tension contributes to clustering of neurotransmitter vesicles at presynaptic terminals.. Proceedings of the National Academy of Sciences of the United States of America.

[45] Bray D (1979). Mechanical Tension Produced by Nerve-Cells in Tissue-Culture.. Journal of Cell Science.

[46] Bray D (1987). Growth Cones - Do They Pull or Are They Pushed.. Trends in Neurosciences.

[47] Lamoureux P, Buxbaum RE, Heidemann SR (1989). Direct Evidence That Growth Cones Pull.. Nature.

[48] Song S, Sjostrom PJ, Reigl M, Nelson S, Chklovskii DB (2005). Highly nonrandom features of synaptic connectivity in local cortical circuits.. Plos Biology.

[49] Blinder P, Cove J, Foox M, Baranes D (2008). Convergence among Non-Sister Dendritic Branches: An Activity-Controlled Mean to Strengthen Network Connectivity.. Plos One.

[50] Wen Q, Chklovskii DB (2005). Segregation of the brain into gray and white matter: A design minimizing conduction delays.. Plos Computational Biology.

[51] Itzkovitz S, Baruch L, Shapiro E, Segal E (2008). Geometric constraints on neuronal connectivity facilitate a concise synaptic adhesive code.. Proceedings of the National Academy of Sciences of the United States of America.

[52] Whitesides GM, Grzybowski B (2002). Self-assembly at all scales.. Science.

[53] Lumelsky N, Blondel O, Laeng P, Velasco I, Ravin R (2001). Differentiation of embryonic stem cells to insulin-secreting structures similar to pancreatic islets.. Science.

[54] Petermann T, Thiagarajan TC, Lebedev MA, Nicolelis MAL, Chialvo DR (2009). Spontaneous cortical activity in awake monkeys composed of neuronal avalanches.. Proceedings of the National Academy of Sciences of the United States of America.

[55] Kaschube M, Schnabel M, Lowel S, Coppola DM, White LE (2010). Universality in the Evolution of Orientation Columns in the Visual Cortex.. Science.

[56] Shepherd GMG, Stepanyants A, Bureau I, Chklovskii D, Svoboda K (2005). Geometric and functional organization of cortical circuits.. Nature Neuroscience.

[57] Murre JMJ, Sturdy DPF (1995). The Connectivity of the Brain - Multilevel Quantitative-Analysis.. Biological Cybernetics.

[58] Yizhar O, Fenno LE, Prigge M, Schneider F, Davidson TJ (2011). Neocortical excitation/inhibition balance in information processing and social dysfunction.. Nature.

[59] Adesnik H, Scanziani M (2010). Lateral competition for cortical space by layer-specific horizontal circuits.. Nature.

[60] Shein Idelson M, Ben-Jacob E, Hanein Y (2010). Innate Synchronous Oscillations in Freely-Organized Small Neuronal Circuits.. Plos One.

[61] Yu Y-C, Bultje RS, Wang X, Shi S-H (2009). Specific synapses develop preferentially among sister excitatory neurons in the neocortex.. Nature.

[62] Silva GA, Czeisler C, Niece KL, Beniash E, Harrington DA (2004). Selective differentiation of neural progenitor cells by high-epitope density nanofibers.. Science.

[63] Taylor AM, Dieterich DC, Ito HT, Kim SA, Schuman EM (2010). Microfluidic Local Perfusion Chambers for the Visualization and Manipulation of Synapses.. Neuron.

[64] Pautot S, Wyart C, Isacoff EY (2008). Colloid-guided assembly of oriented 3D neuronal networks.. Nature Methods.

[65] Silva GA (2006). Neuroscience nanotechnology: Progress, opportunities and challenges.. Nature Reviews Neuroscience.

[66] Bullmore E, Sporns O (2009). Complex brain networks: graph theoretical analysis of structural and functional systems.. Nature Reviews Neuroscience.

[67] Rabinovich MI, Varona P, Selverston AI, Abarbanel HDI (2006). Dynamical principles in neuroscience.. Reviews of Modern Physics.

[68] Jang KJ, Kim MS, Feltrin D, Jeon NL, Suh KY (2010). Two Distinct Filopodia Populations at the Growth Cone Allow to Sense Nanotopographical Extracellular Matrix Cues to Guide Neurite Outgrowth.. Plos One.

[69] Li Y, Yuan B, Ji H, Han D, Chen S (2007). A method for patterning multiple types of cells by using electrochemical desorption of self-assembled monolayers within microfluidic channels.. Angew Chem Int Ed Engl.

[70] Liu DB, Xie YY, Shao HW, Jiang XY (2009). Using azobenzene-embedded self-assembled monolayers to photochemically control cell adhesion reversibly.. Angew Chem Int Ed Engl,.

[71] Jiang XY, Ferrigno R, Mrksich M, Whitesides GM (2003). Electrochemical desorption of self-assembled monolayers noninvasively releases patterned cells from geometrical confinements.. Journal of the American Chemical Society.

[72] Dertinger SKW, Jiang XY, Li ZY, Murthy VN, Whitesides GM (2002). Gradients of substrate-bound laminin orient axonal specification of neurons.. Proceedings of the National Academy of Sciences of the United States of America.

[73] Peng YR, He S, Marie H, Zeng SY, Ma J (2009). Coordinated Changes in Dendritic Arborization and Synaptic Strength during Neural Circuit Development.. Neuron.

[74] Yuste R, Ikegaya Y, Aaron G, Cossart R, Aronov D (2004). Synfire chains and cortical songs: Temporal modules of cortical activity.. Science.

