# Supplementary figures and images for: Self-Organizing Circuit Assembly through Spatiotemporally Coordinated Neuronal Migration within Geometric Constraints
Source: PLoS One. 2011 Nov 22;6(11):e28156. doi: 10.1371/journal.pone.0028156 (PMC3222678; doi:10.1371/journal.pone.0028156)

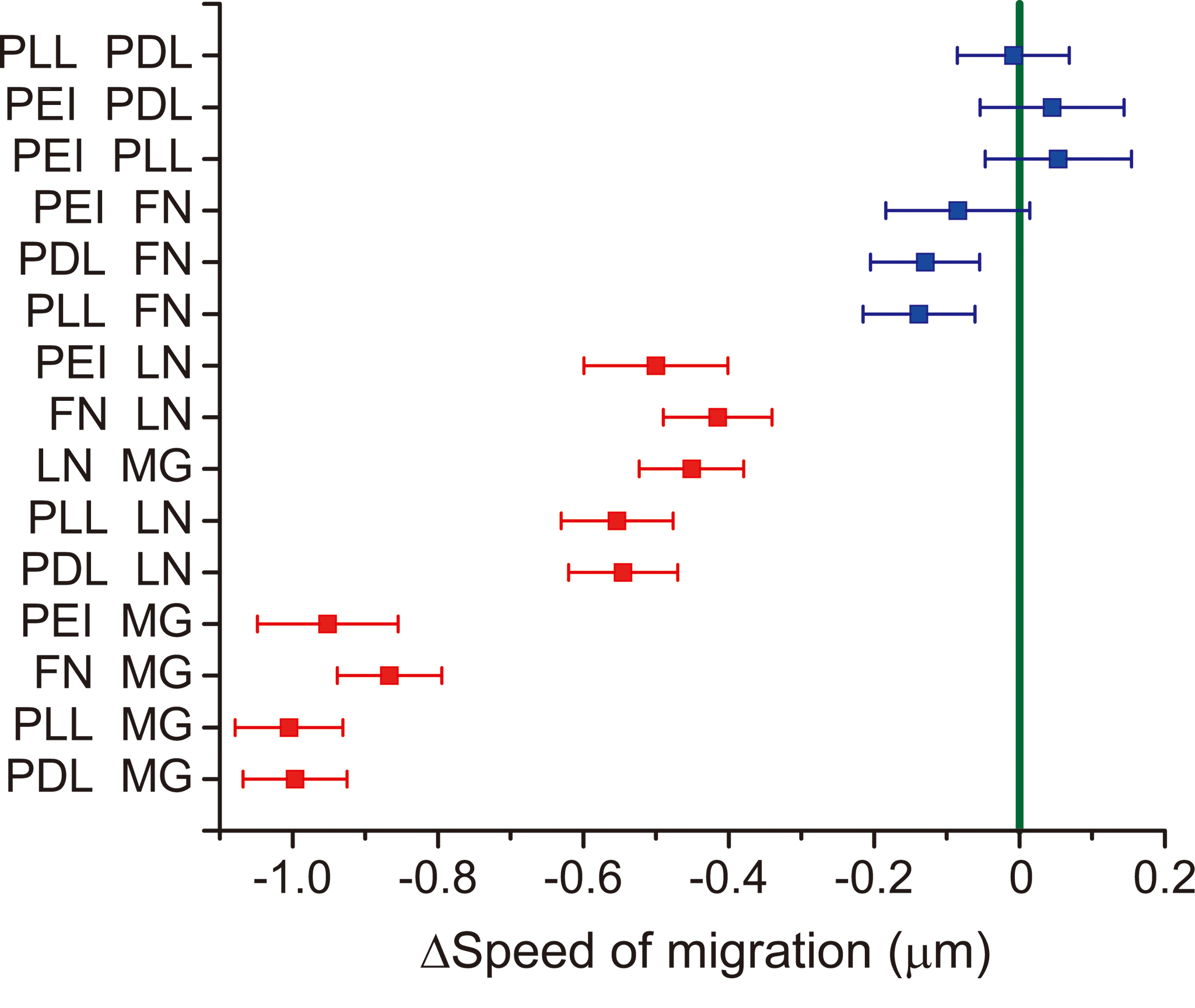

Supplement: Figure S1 — Multiple Comparisons test on the speed of neuronal migration on gold substrates with various surface coatings. Red error bars denote significant difference (P<0.05), while blue error bars denote non-significant difference. The speed of neuronal migration on MG coating is significantly higher than any other coatings (One-way ANOVA followed by Tukey's post hoc test). (TIF) [file pone.0028156.s001.tif]

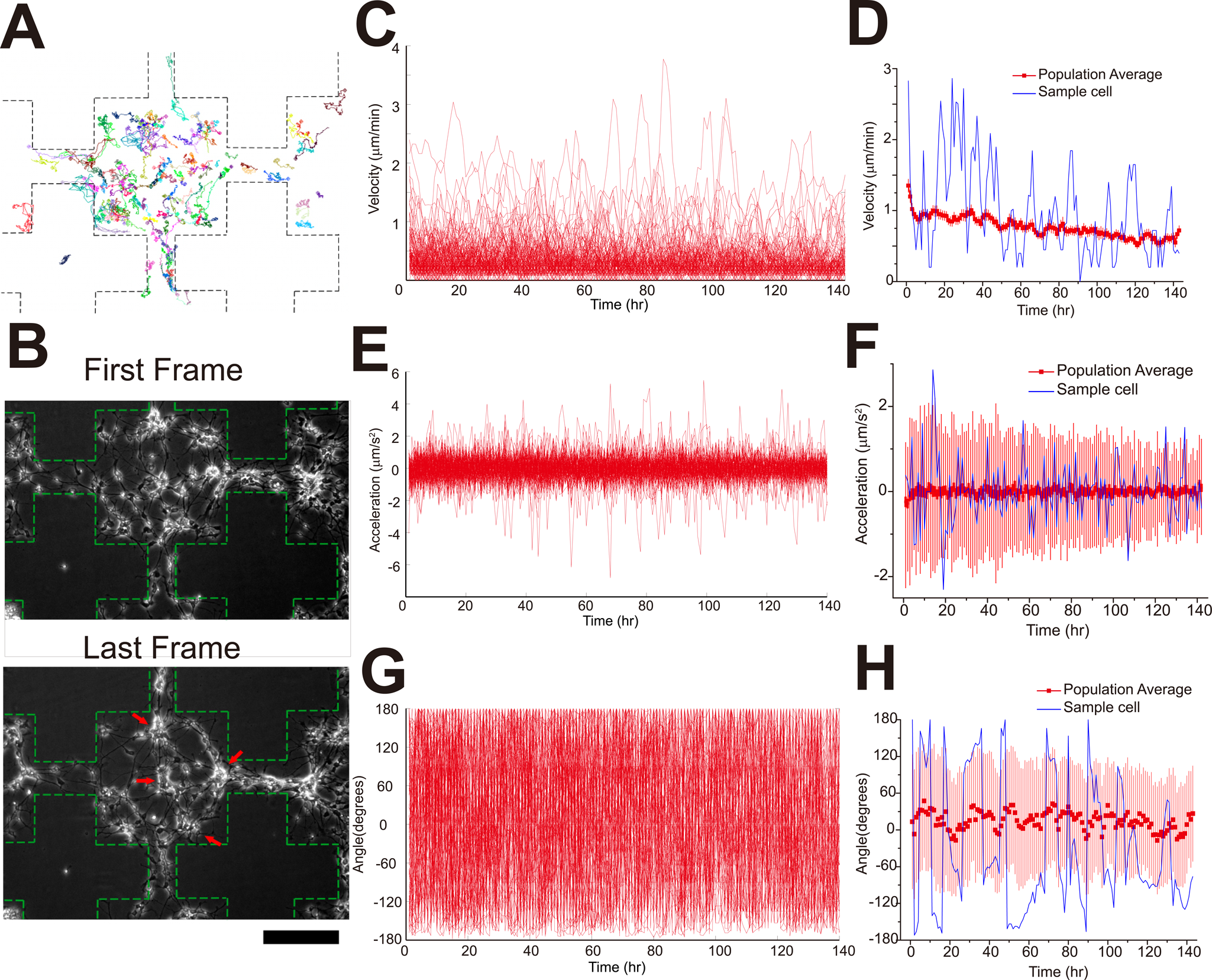

Supplement: Figure S2 — Neuronal migration in confined space at 0 DIV. A, The migration trajectories of 135 neurons located on a square-shaped network traced with time-lapse imaging for 12 hours with an interval of 5 minutes between each frame. Each colored line corresponds to an individual neuron. Green dashes depict the geometry of the geometric constraint. B, Phase-contrast image of the first and the last frame, red arrows point to emerging clusters. C - H, Motion analysis of the neurons shown in panel A over 12 hr in terms of velocity, acceleration and angle. C, E, G are superimposed velocity, acceleration and angle of all 135 neurons in the network over time. Huge variations in migration capability between neurons in terms of velocity (C), acceleration (E) and angle (G) can be seen. D, F, H are density plots showing the mean (red box) and S.E.M. (line), with a superimposed blue curve showing the behavior of a randomly chosen sample neuron. A gradual decrease of velocity as time elapses can be seen in panel D (Spearman's correlation coefficient is 0.5332, P<0.0001). Sharp bursts in speed take place randomly. While the mean values of acceleration are largely constant, the S.E.M. demonstrate a gradual narrowing over time (panel F, Spearman's correlation coefficient is 0.4582, P<0.0001). The envelope of angle ebb and flow (panel G), but the S.E.M. gradually decreases (panel H, Spearman's correlation coefficient is 0.3213, P<0.0001). Meanwhile, the behavior of a single neuron is characterized by random fluctuations. Scale bar: 200 µm. (TIF) [file pone.0028156.s002.tif]

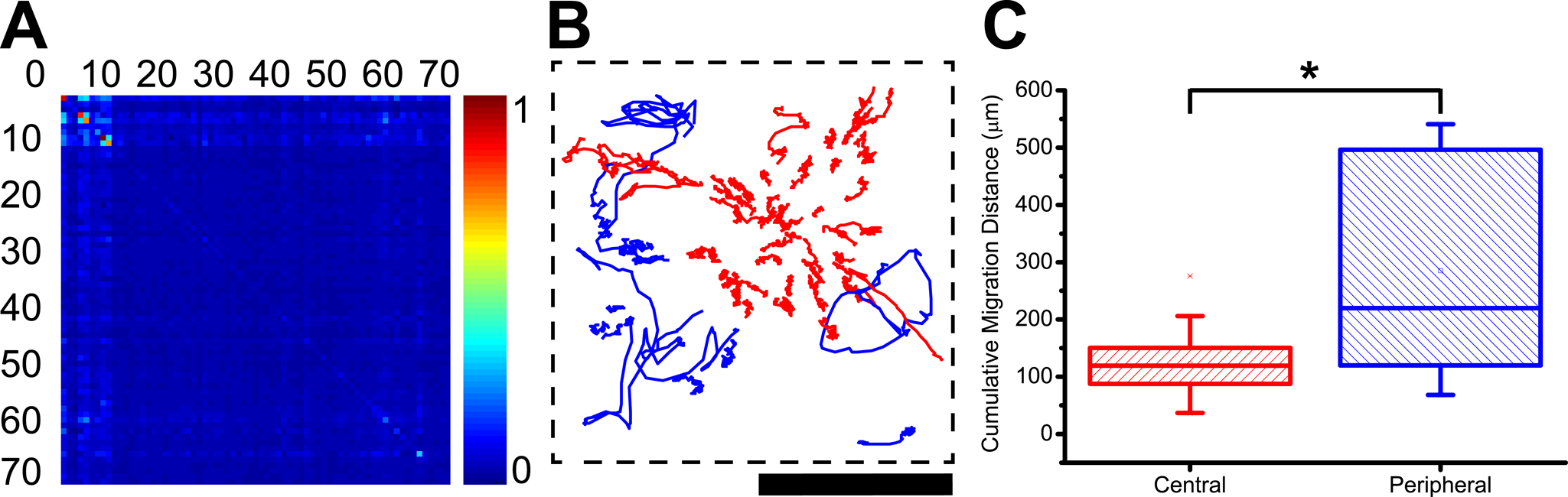

Supplement: Figure S3 — Heterogeneity of collective migration. A, Color-coded normalized covariance matrix for the velocities of all cells in Figure 3E. The numbers are the serial numbers of the cells. The color of each dot in the matrix represents the covariance coefficient between the two cells designated by the horizontal and vertical index of the matrix respectively. The color bar relates different colors to the covariance coefficients (between 0 and 1). B, The projection of the three-dimensional figure in Figure 3E onto the horizontal plane. We categorized the cells according to their covariance coefficients (panel A) through k-means clustering, cells with larger covariance coefficients are labeled blue while the rest are labeled red. The two populations of neurons exhibit two modes of migration. Neurons with red-colored trajectories are coordinated by the centripetal movement, while neurons with blue-colored trajectories show edge-philic movement. C, Box chart showing the cumulative distance that the red and blue group of neurons travel (* P<0.001, Kolmogorov-Smirnov test, Central: n = 56, Peripheral: n = 13). Scale bar: 200 µm. (TIF) [file pone.0028156.s003.tif]

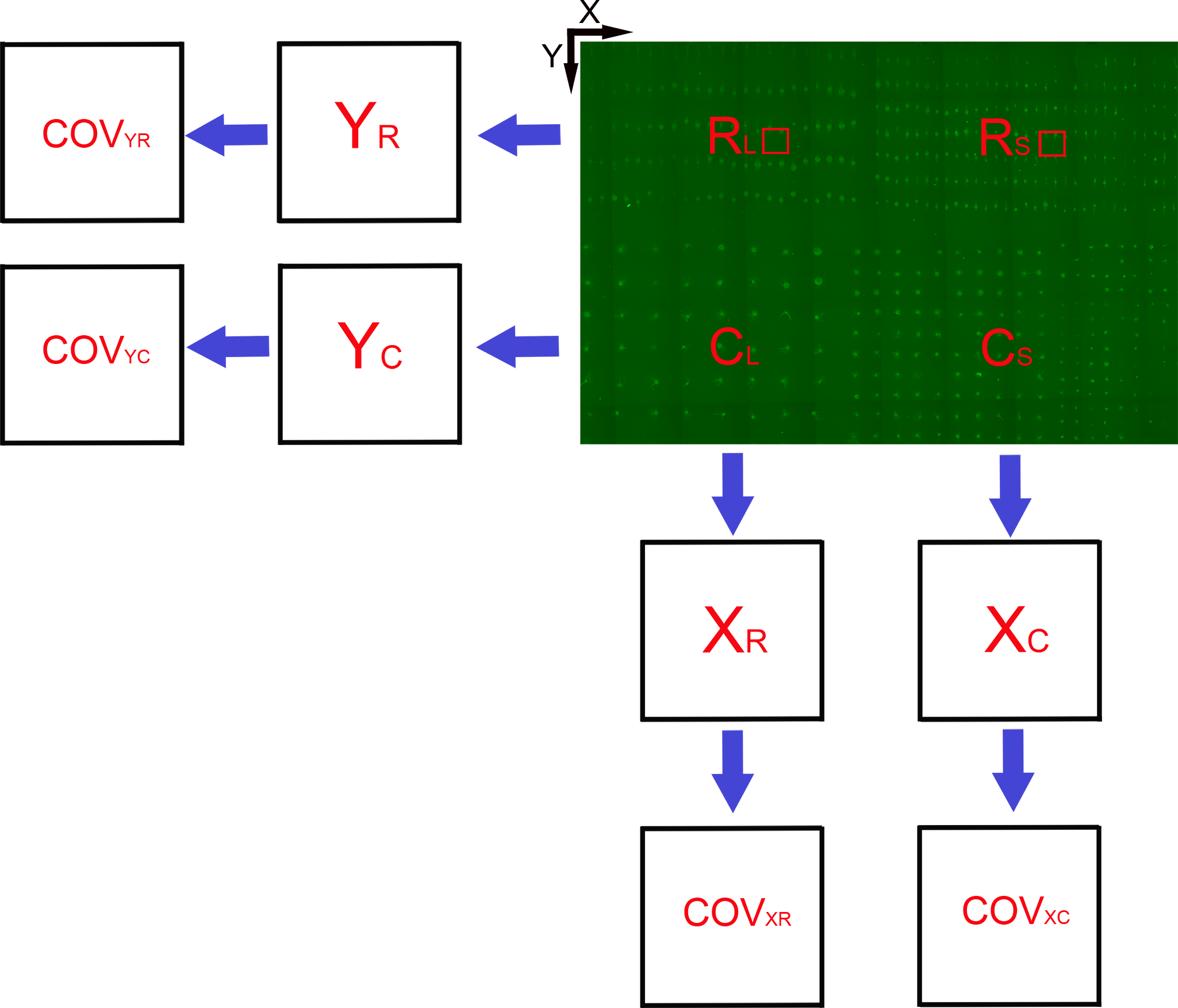

Supplement: Figure S4 — Data extraction strategy for Figure 4F and 4G . The X and Y position of each centralized cluster (shown as a green spot in the array) in the rectangular and circular array of geometric constraints are calculated and converted into matrices. The covariance matrices are then calculated according to the position matrices. (TIF) [file pone.0028156.s004.tif]

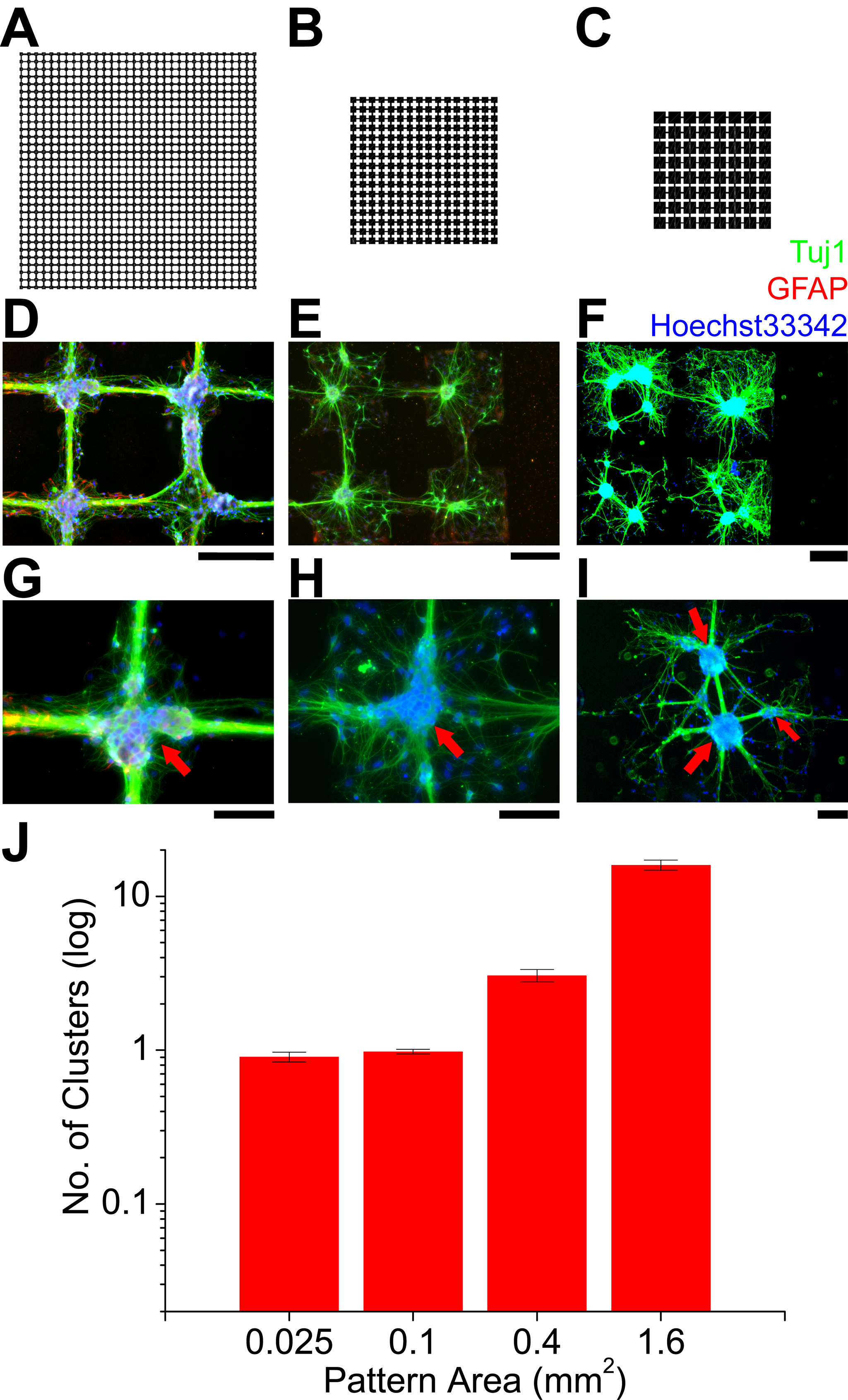

Supplement: Figure S5 — Critical scale determines network structure. Clustering affects neuronal connectivity, immunocytochemistry photomicrographs of geometric constraints shown in A - C. D - F are part of the network composed of four units. G - I immunocytochemistry of a part of the network and an individual unit. Tuj1 (green) is the neuronal marker. J, Emergence of mono-cluster is size dependent. The number of clusters on a geometric constraint increases as the sizes of the square-shaped geometric constraints expands as a geometric progression (n = 5, 10 - 15 data points for each size of geometric constraint). Scale bar: D - F, 250 µm; G - I, 100 µm. (TIF) [file pone.0028156.s005.tif]

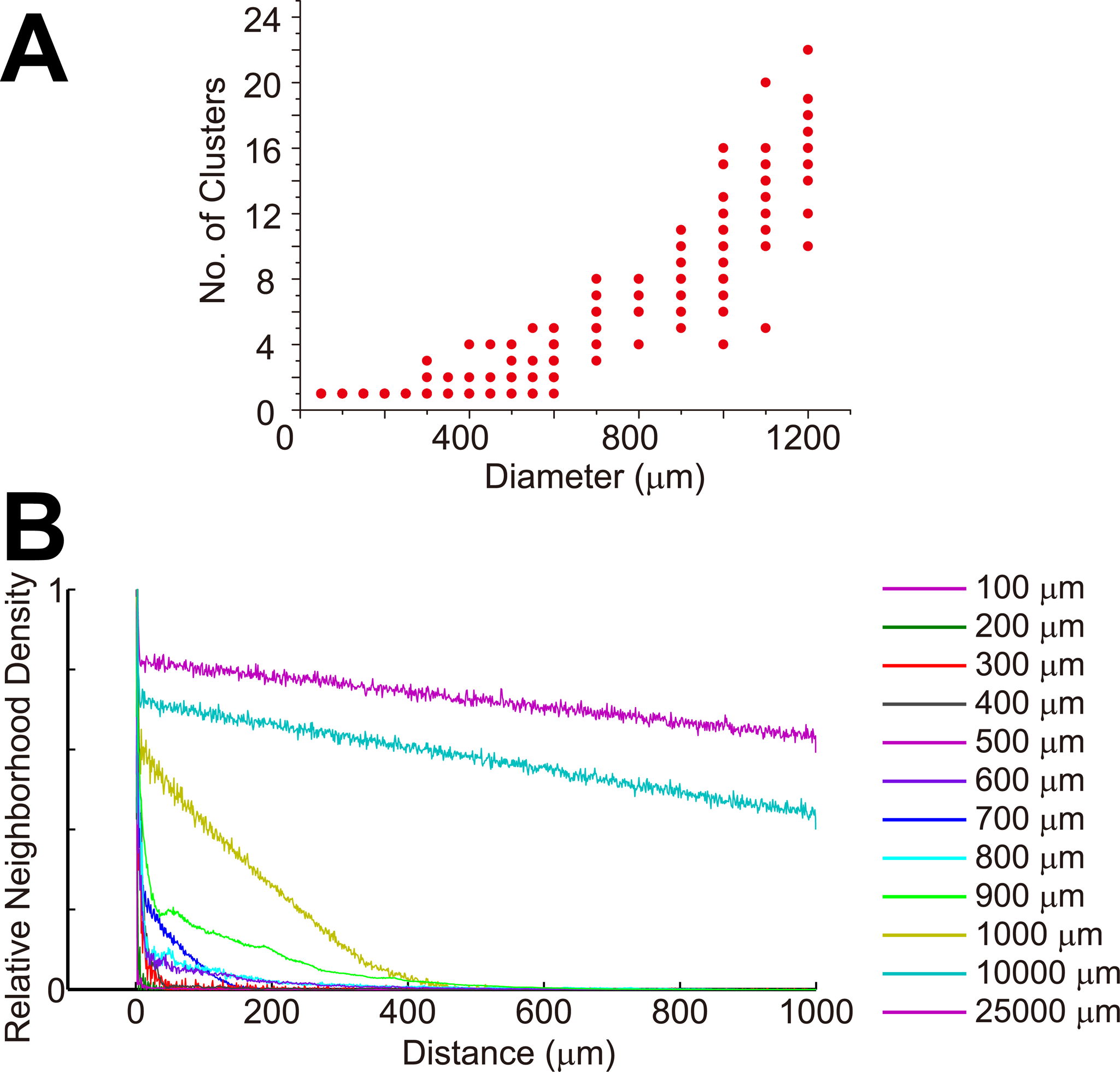

Supplement: Figure S6 — A, Original data points for Figure 6A. Note that each red dot could be many overlapping data points, such as those of 50 to 250 µm where mono-clusters are consistently observed. B, Normalized relative neighborhood density of geometric constraints of various sizes. The horizontal axis is shifted rightward to show the sharp peaks at zero. See Materials and Methods for details. (n = 3, 2000 to 5000 data points for each size of geometric constraint.) (TIF) [file pone.0028156.s006.tif]

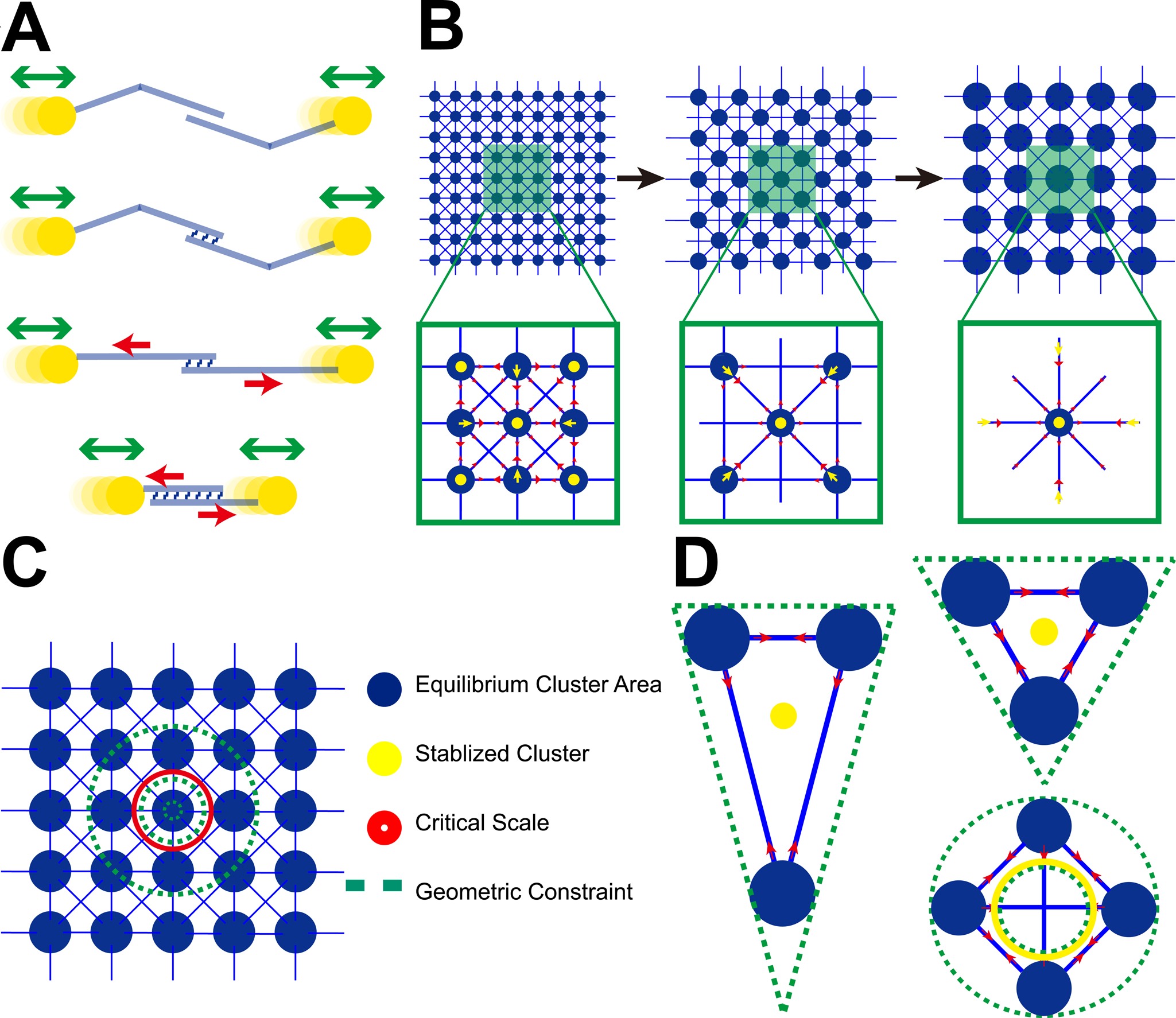

Supplement: Figure S7 — Working model for geometric control over coordinated collective migration via mechanical coupling. A, Fasciculation and tension generation between two adjacent neurons. Contact of adjacent neurites (light blue) facilitates adhesion (deep blue velcro) mediated neurite fasciculation, while the somata (yellow balls) are still subject to active migration. The neurites are then subject to tension force (red arrow), possibly caused by the development of neurite fasciculation. Finally, the fasciculated neurites under tension drag the somata closer. B, Formation of larger clusters on a random network composed of small clusters (dark blue circles) fully connected through fasciculated neurites (light blue lines). The break of symmetry on a neurite will result in directed forces along a neurite, leading to somata migration. C, Size dependent difference in network structure. On very small circuits (dashed circle with smallest diameter), there is no cluster. On circuit above critical scale (dashed circle with largest diameter) there would be multi-clusters. Spatiotemporally coordinated migration and mono-cluster appears when the geometry of the circuit falls in between (dashed circle with medium-sized diameter). The blue balls are stabilized clusters under equilibrium in the absence of geometric constraints. The size of the geometric constraint (green dashes) with respect to the critical scale (red circle) determines the potential number of clusters the circuit would host. D, Geometry regulates cluster location. Filled blue circles represent sub-clusters. Blue lines represent fasciculated neurites between the clusters. Red arrows represent traction forces exerted onto the clusters. Filled yellow circles (and yellow ring in the case of concentric ring) represent final locations of clusters. Green dashed lines are the boundaries of the geometric constraints. The centers of the clusters are located on the geometric centers. When the centripetal movement is blocked by a concentric r [file pone.0028156.s007.tif]
